# Effect of soluble fiber on blood pressure in adults: a systematic review and dose–response meta-analysis of randomized controlled trials

**DOI:** 10.1186/s12937-023-00879-0

**Published:** 2023-10-13

**Authors:** Abed Ghavami, Sara Banpouri, Rahele Ziaei, Sepide Talebi, Mahdi Vajdi, Elyas Nattagh‐Eshtivani, Hanieh Barghchi, Hamed Mohammadi, Gholamreza Askari

**Affiliations:** 1 Department of Clinical Nutrition, School of Nutrition and Food Science, Isfahan University of Medical Sciences, Isfahan, Iran; 2 Nutrition and Metabolic Diseases Research Center, Ahvaz Jundishapur University of Medical Sciences, Ahvaz, Iran; 3 Department of Community Nutrition, School of Nutrition and Food Science, Isfahan University of Medical Sciences, Isfahan, Iran; 4 Department of Clinical Nutrition, School of Nutritional Sciences and Dietetics, Tehran University of Medical Sciences, Tehran, Iran; 5 Student Research Committee, Department of Community Nutrition, School of Nutrition and Food Science, Isfahan, Iran; 6 Department of Nutrition, Food Sciences and Clinical Biochemistry, School of Medicine, Social Determinants of Health Research Center, Gonabad University of Medical Science, Gonabad, Iran; 7 Student Research Committee, Mashhad University of Medical Sciences, Mashhad, Iran

**Keywords:** Soluble fiber, Blood pressure, Meta-analysis, Systematic review

## Abstract

**Objectives:**

Pervious epidemiologic evidence indicates that soluble fiber is protective against hypertention: however, randomized controlled trials (RCTs) have presented varying results. In the present study, we aimed to conduct a systematic review and dose–response meta-analysis to summarize published RCTs which assess the effect of soluble fiber supplementation on systolic blood pressure (SBP) and diastolic blood pressure (DBP).

**Methods:**

Scopus, PubMed, and ISI Web of Sciences were searched to identify relevant studies up to Aug 2022. We estimated the change in blood pressure for each 5 g/d increment in soluble fiber supplementation in each trial and then calculated the weighted mean difference (WMD) and 95%CI using a random-effects model. We estimated dose-dependent effects using a dose–response meta-analysis of differences in means. The risk of bias for study was assessed using the Cochrane tool. Publication bias was evaluated via funnel plot and Begg’s test and Egger's test.

**Results:**

Eighty-three eligible studies with total sample size of 5,985 participants were included in the meta-analysis. Soluble fiber supplementation significantly decreased SBP (WMD: -1.36 mmHg, 95% CI: -2.13 to -0.60, *P* < 0.001; I^2^ = 47.1%, *P* < 0.001) and DBP (WMD: -0.72 mmHg, 95% CI: -1.26 to -0.18, *P* = 0.009; I^2^ = 45.4%, *P* < 0.001). Each 5 g/d increment in soluble fiber supplementation had a significant reduction in SBP (WMD: -0.54 mmHg; 95%CI: -0.86, -0.22, *P* = 0.001; I^2^ = 52.2, P_het_ < 0.001) and DBP (WMD: -0.28 mmHg; 95%CI: -0.49, -0.80, *P* = 0.007; I^2^ = 43.1%, P_het_ < 0.001). The levels of SBP decreased proportionally with the increase in soluble fiber supplementation up to 20 g/d (MD_20g/d_: -1.79 mmHg, 95%CI: -2.86, -0.71).

**Conclusion:**

Current evidence indicated the beneficial effect of soluble fiber supplementation on blood pressure. Our findings suggest that soluble fiber supplementation could contribute to the management of hypertension and the reduction of cardiovascular disease risk.

**Supplementary Information:**

The online version contains supplementary material available at 10.1186/s12937-023-00879-0.

## Introduction

Hypertension or high blood pressure (BP) is one of the most prevalent and severe vascular diseases. In 2015, 8.5 million deaths were attributable to high blood pressure, mainly happen in low-income and middle-income countries [1]. As a widely accepted fact, untreated or poorly controlled high BP is one of the most important factors contributed to cardiovascular disease (CVD), stroke, eye problem, heart failure, chronic kidney disease, and increases health-care costs all over the world [1]. Therefore, anti-hypertensive approaches are of great importance to improve the health status of hypertensive patients. Based on current guidelines for hypertension management, lifestyle and dietary modifications should be considered as first-line anti-hypertensive strategies [2]. Recent data propose that a high content of dietary fiber is helpful for people with hypertension [3]. However, it is often difficult for people to consume sufficient fiber, from diet alone, to achieve the desired results. Therefore, fiber supplementation would be an opportunity and cost-effective approach to consume enough fiber to improve hypertension and its associated cardiovascular complications. Dietary fibers are generally of two types based on their solubility (soluble and insoluble) and their physicochemical properties effect therapeutic intake effects [4].

Previous review studies have reported the beneficial effects of soluble fiber such as psyllium, inulin, glucomannan, guar gum, and pectin in reducing the risk of chronic diseases such as CVDs, obesity, diabetes, and hyperinsulinemia [4, 5]. However, studies on anti-hypertensive properties of soluble fiber are controversial and inconsistent. Some clinical trials suggested a significant effect of soluble fiber intake on high BP. In contrast, other did not support such result. For example, a study found that consumption of soluble fiber has beneficial effects on BP [6]. On the contrary, results of another study did not show any protective effect of soluble fiber intake over six week on BP [7].

Some reviews examined the effect of soluble and insoluble-fiber supplementation on BP [8–10]. However, they have examined a considerable low number of trials and the results of them are inconsistent and after these meta-analyses, several randomized controlled trials (RCTs) have been published which may change the result of previous meta-analyses. Therefore, the current study aimed to perform a comprehensive systematic review and dose–response meta-analysis of available RCTs to evaluate the efficacy of soluble fiber consumption on BP in adults.

## Methods

This systematic review and meta-analysis followed the PRISMA (Preferred Reporting Items for Systematic Review and Meta-analysis) guideline [11].

The PICOS criteria were applied to formulate the question: Population (adults aged 18 and older), Intervention (soluble fiber supplementation), Compression (not using soluble fiber supplementation), Outcome (changes of SBP and DBP), and Study design (parallel and crossover clinical trials).

### Search strategy and eligibility criteria

Systematic literature searches for published articles were conducted in PubMed/Medline, Scopus and Web of Science without time and language restrictions through Aug 2022. A comprehensive description of the search strategy is provided in Table S1. In addition, we performed a reference list check of relevant articles, reviews, and meta-analyses to avoid missing any relevant literature.

We included studies based on the following inclusion criteria: (1) RCTs with either parallel or crossover design; (2) studies conducted on the adult population (≥ 18 years); (3) studies that involved comparison groups who received either a placebo without soluble fiber or an isolated soluble fiber treatment as part of controlled consumption studies or ad libitum supplementation interventions that were conducted in free-living subjects; and (4) studies that reported adequate baseline and follow up data in both treatment and control groups.

We excluded observational studies, animal or in-vitro studies, and those conducted on children, pregnant, or lactating women. In addition, studies without a control group, those that reported insufficient data on the selected outcomes in the soluble fiber or control groups, and trials that examined the effect of soluble fiber in combination with other components were not included. Additionally, trials that included soluble fiber supplementation as part of a dietary mixture or mixed within a dietary substance were excluded from analysis if the effects of soluble fiber alone could not be determined.

### Screening and data extraction

Abstracts and full texts of qualified studies were screened independently by two reviewers (SB and AG), who were blinded to the studies' authors or results. A chief reviewer (GA) was consulted to reach a consensus when necessary.

The following information was extracted from each eligible trial: first author's name, year of publication, study location, length of intervention, study design, characteristics of enrolled participants (numbers, mean age, sex, and health status of them), and soluble fiber intervention (form of adminstration, type, dosage, fermentability and viscosity), comparator and background diet. The mean and standard deviation (SD) of SBP and DBP before and after intervention alongside changes between baseline and post intervention were extracted. The mean and SD were extracted or calculated from available reported data [reported as 95% confidence intervals (CI), standard error (SE), median and (interquartile range (IQR)] using a standardized formula [12] at baseline and at the end of the respective treatment periods and were incorporated in the meta-analysis. When outcome measures were reported in figures/plots alone (e.g., no mean data reported), WebPlotDigitzer 4.5 was utilized to estimate equivalent numerical values. For multi-arm trials, intervention groups were isolated to determine the independent effects of soluble fiber supplementation across treatments. Paired analyses were conducted for all crossover trials [13].

### Risk of bias and certainty of evidence

Quality assessment was undertaken to assess the rigor of RCTs as determined by the risk of bias utilizing the Cochrane Risk of Bias Assessment Tool Evaluation of RCT quality (bias) was based on seven domains: participant randomization sequence generation, supplement allocation concealment, blinding of participants and research personnel, blinding of outcome assessment, incomplete outcome data, selective outcome reporting, and other bias sources. The risk of bias for each study was then rated as low (adequate information provided), unclear (if certain information was unclear or indeterminate), and high (if there was a serious concern in the criteria). The overall quality of studies was graded as *good* if there were low risk of bias for more than two items, *fair* if there were low risk of bias for two items and *poor* if there was a low risk of bias for less than two items [14].

The Grading of Recommendations Assessment, Development and Evaluation [15] method was employed to evaluate the certainty of the evidence for outcomes. The quality of the assessed evidence was rated as high, moderate, low, and very low. High grades suggest high confidence that the actual effect is commensurate with the estimated effect. Moderate grades suggest that the actual effect is likely to be close to the estimate of the effect; however, there exists a small possibility of substantial differences. A low grade suggests a greater likelihood that the true effect may be substantially different from the estimate of the effect, and very low grades suggest the true effect is likely different from the estimated effect. Further, RCTs with an initial high quality of evidence evaluation may be downgraded based on study limitations, including risk of bias inconsistency (substantial unexplained heterogeneity, I^2^ > 50%; *p* < 0.05) and imprecision (95% CI for effect estimates are wide or cross the minimally significant threshold difference for clinical benefit). Minimal thresholds for clinically important changes and consider indirectness of outcomes (primary outcomes presented are surrogate rather than patient-important outcomes [15] and other considerations (publication bias and dose–response gradient usage).

### Data synthesis and statistical analysis

The mean and SD changes of SBP and DBP were used to calculate the pooled effect sizes. Studies that did not report the SD of the mean differences in each group required manual calculation as follows [16];

$${SD}_{change}=\sqrt{{SD}_{baseline}^{2}+{SD}_{final}^{2}-2\times R\times {SD}_{baseline}\times {SD}_{final}}$$

where R represents a correlation coefficient of 0.5 [17]. Because pretest to posttest R was not reported in RCTs, an R-value of 0.5 was utilized throughout this meta-analysis [18]. Also, we conducted sensitivity analyses using different correlation values (0.25 and 0.75) and report and interpretation meta-analysis results. Due to the observed variation in study treatments and protocol, a random-effects model was used to estimate mean difference (MD) and 95% confidence interval (CIs) [18].

We conducted meta-regressions to assess outcomes in relation to the following prespecified factors: intervention duration, daily dose of soluble fiber, soluble fiber category (type, fermentability and viscosity), gender, mean baseline BMI, mean age, and mean baseline SBP and DBP and health status. Factors were selected on the basis of the likelihood of influence on outcomes of interest. The intervention duration was defined as the time period (weeks) when participants received the treatment or placebo; the soluble fiber dose was defined as daily grams of fiber treatment as provided during the intervention; and the categorization of soluble fibers was based on their physicochemical properties (type, viscosity, and fermentability) [19]. Mean BMI was categorized to obese (≥ 30 and non-obese < 30), mean baseline SBP and DBP was categorized to hypertention (SBP ≥ 130 mmHg and DBP ≥ 80) [20], classified trials based on mean age (equal and more than 50 years and bellow 50 years), and according to different health status (individual with hypertension, metabolic syndrome, hypercholesterolemia, hyperlipidemia, diabetes, overweight-obese, and otherwise healthy).

We assessed the between-study heterogeneity of soluble fiber intervention effects using the I^2^ statistic. The following I^2^ interpretive categories were used: bellow I^2^ < 50% was considered “moderate”, (50% <  = I^2^ <  = 75%), was considered “substantial” heterogeneity and “considerable” heterogeneity (I^2^ >  = 75%) [18]. Sensitivity analyses assessed the impact of each study on the pooled effect size [21]. The risk of publication bias was assessed using visual inspection of the funnel plot and Begg’s test and Egger's test [22]. Based on Crippa and Orsini's method, the mean and corresponding SD of change in liver function test, and the number of participants in each study arm, was used to conduct a random-effects model for each 5 g/d increase in soluble fiber supplementation in the intervention group on changes in liver function test [23]. Additionally, we conducted a dose–response meta-analysis to clarify the shape of the effect of different doses of soluble fiber on blood lipids [24]. All statistical tests were performed using the Stata software (Version 17.0, Stata Corp, College Station, TX), and a *P*-value less than 0.05 considered significant.

## Results

### Study selection

**Fig. 1:**
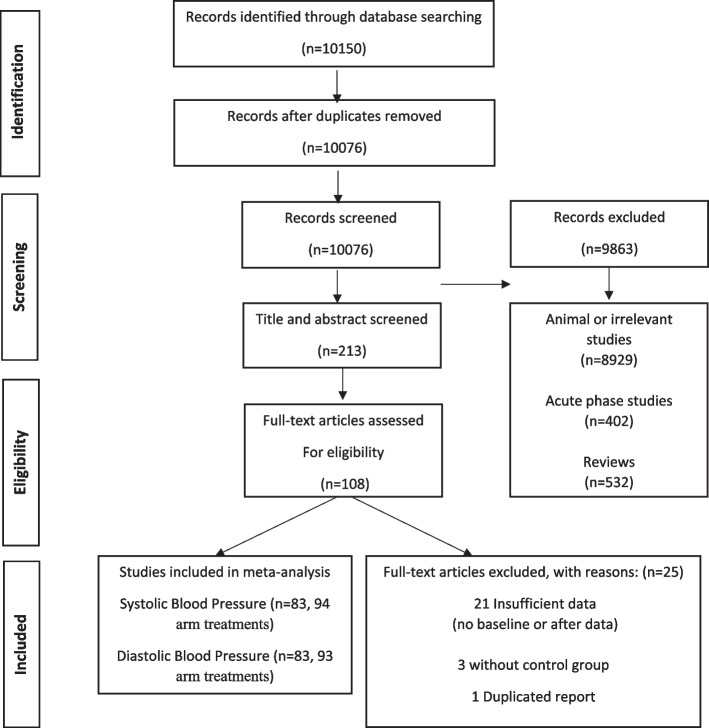
Flowchart diagram for study selection of systematic review

A total of 10,150 records were initially retrieved, of them 74 duplicate publications were removed. After screening, 9863 studies (animal or irrelevant studies (*n* = 8929) and acute phase (*n* = 402) or review (*n* = 532) articles were removed. 106 trials removed from 213 trials which covered blood pressure outcomes. We included 108 articles for further examination of full texts. Of these articles, 25 studies were excluded due to following reasons: without sufficient data for outcomes (baseline or final assessment) (*n* = 20), irrelevant supplementation (*n* = 1), without control group (*n* = 3) and one of them due to duplicate report. Eighty-three eligible trials were included in the final quantitative analysis. Also, except two studies [25, 26] other studies included in dose–response analysis. The flow diagram of detailed steps of the literature search process and excluded studies is illustrated in Fig. 1 and Table S2.

### Study characteristics

The general characteristics of the 83 qualified articles are illustrated in Table S3. Selected eligible trials enrolled 5,985 (3026 intervention and 2959 control) participants with age ranging from 22.9 to 68.3 years old. Most of studies were conducted on hypercholesterolemia, overweight and obese, metabolic syndrome and people with diabetes, healthy and seven studies on hypertensive patients. At the time of recruitment, BMI varied between 21.9 and 37.7 kg/m2. These trials were published from 1988 to 2021 and performed in the different site of the world. The 89, 11 percent of selected studies used a parallel and cross over design respectively. Duration of soluble fiber supplementation varied from 3 to 52 weeks. Daily prescribed dosage of soluble fiber varied between 0.477 and 45 g per day.

### Findings from meta-analysis

Ninety-four eligible effect sizes including a total of 5867 participants (2970 Intervention 2897 Control) examined the effect of soluble fiber supplementation on SBP. Combining their findings based on random-effects model, we found that SBP was significantly reduced in soluble fiber group compared to the control (WMD: -1.36 mmHg, 95% CI: -2.13 to -0.60, *P* < 0.001), including a moderate significant heterogeneity between studies (I^2^ = 47.1%, *P* < 0.001) (Table 1). To find source of heterogeneity, subgroup analysis revealed that duration of intervention (I^2^ = 7.6%, *P* = 0.328), participants gender (I^2^ = 12.6%, *P* = 0.332), baseline SBP of subjects (I^2^ = 21.9%, *P* = 0.091) and health status (I^2^ = 0.0%, *P* = 0.637) were the potential sources of heterogeneity.

**Table 1:** The effect of soluble fiber supplements on blood pressure

		**Pairwise meta-analysis**				**Dose–response meta-analysis**					
	**Studies, *n***	**MD (95% CI)**	***P value***	***I***^***2***^**, %**	***P***_**heterogeneity**_	**Dose, g/d**	**Studies, *n***	**WMD (95% CI)**	***P* value**	***I***^***2***^**, %**	***P***_**heterogeneity**_
SBP	94	-1.36 (-2.13, -0.60)	< 0.001	47.1	< 0.001	5	92	-0.54 (-0.86, -0.22)	0.001	52.2	< 0.001
DBP	93	-0.72 (-1.26, -0.18)	0.009	45.4	< 0.001	5	91	-0.28 (-0.49, -0.80)	0.007	43.1	< 0.001

*Abbreviations: MD* Mean Difference, *CI* Confidence Interval

Ninety-three qualified effect sizes including a total 5867 participants (2970 Intervention 2897 Control) subjects, reported DBP as their outcome. After pooling these studies based on random-effects model, we found a significant reduction in DBP following soluble fiber supplementation (WMD: -0.72 mmHg, 95% CI: -1.26 to -0.18, *P* = 0.009), with a moderate significant between-study heterogeneity (I^2^ = 45.4%, *P* < 0.001) (Table 1). However, participants’ mean age (I^2^ = 21.2%, *P* = 0.102), subjects mean BMI (I^2^ = 4.3%, *P* = 0.386), participants gender (I^2^ = 25.2%, *P* = 0.228), and their health status (I^2^ = 0.0%, *P* = 0.853) were responsible, at least to some extent, for between-study heterogeneity.

Table S4 shows sensitivity analyses in which we used different correlation coefficients (0.25 and 0.75) for paired analyses to calculate standard errors. The findings for every result were unchanged regardless of the correlation coefficients.

#### Subgroup analysis

Findings from the subgroup analyses are outlined in Table S5. After categorizing studies on the basis of participant mean age, subjects older than 50 years (WMD:-2.16 mmHg, 95% CI: -3.06 to -1.27; *P* < 0.001), health status people with diabetes (WMD: -2.50 mmHg, 95% CI: -4.84 to -0.16; *P* = 0.036), metabolic syndrome (WMD: -2.49 mmHg, 95% CI: -4.29 to -0.68; *P* = 0.007), and patient with hypertension (WMD: -2.91 mmHg, 95% CI: -4.08 to -1.73; *P* < 0.001) and fermentability of soluble fiber, fermented fiber (WMD: -1.38 mmHg, 95% CI: -2.16 to -0.59; *P* = 0.001) SBP was significantly decreased.

The reduction in DBP after soluble fiber supplementation was remarkable in subjects with mean BMI > 30 years old (WMD: -1.62 mmHg, 95% CI: -2.98 to -0.27; *P* = 0.019), trials lasted 8 weeks (WMD: -0.91 mmHg, 95% CI: -1.57 to -0.23; *P* = 0.008), trial using fermentable fiber (WMD: -0.81 mmHg, 95% CI: -1.41 to -0.21; *P* = 0.008) and hypertension population (WMD: -2.10 mmHg, 95% CI: -3.16 to -1.03; *P* < 0.001).

According to subgroup analysis based on type of fiber supplemented, SBP and DBP had a larger reduction in guar gum subset (WMD: -2.25 mmHg, 95% CI: -4.14 to -0.37; *P* = 0.019) and inulin (WMD: -3.20 mmHg, 95% CI: -5.19 to -1.22; *P* = 0.002), respectively.

#### Dose–response relation between doses of soluble fiber supplementation and blood pressure parameter

Each 5 g/d increment in soluble fiber consumption significantly reduced SBP (WMD: -0.54; 95%CI: -0.86, -0.22, *P* = 0.001; I^2^ = 52.2, P_het_ < 0.001; *n* = 92 trials; Table 1) and DBP (WMD: -0.28; 95%CI: -0.49, -0.80, *P* = 0.007; I^2^ = 43.1%, P_het_ < 0.001; *n* = 91 trials; Table 1).

**Fig. 2:**
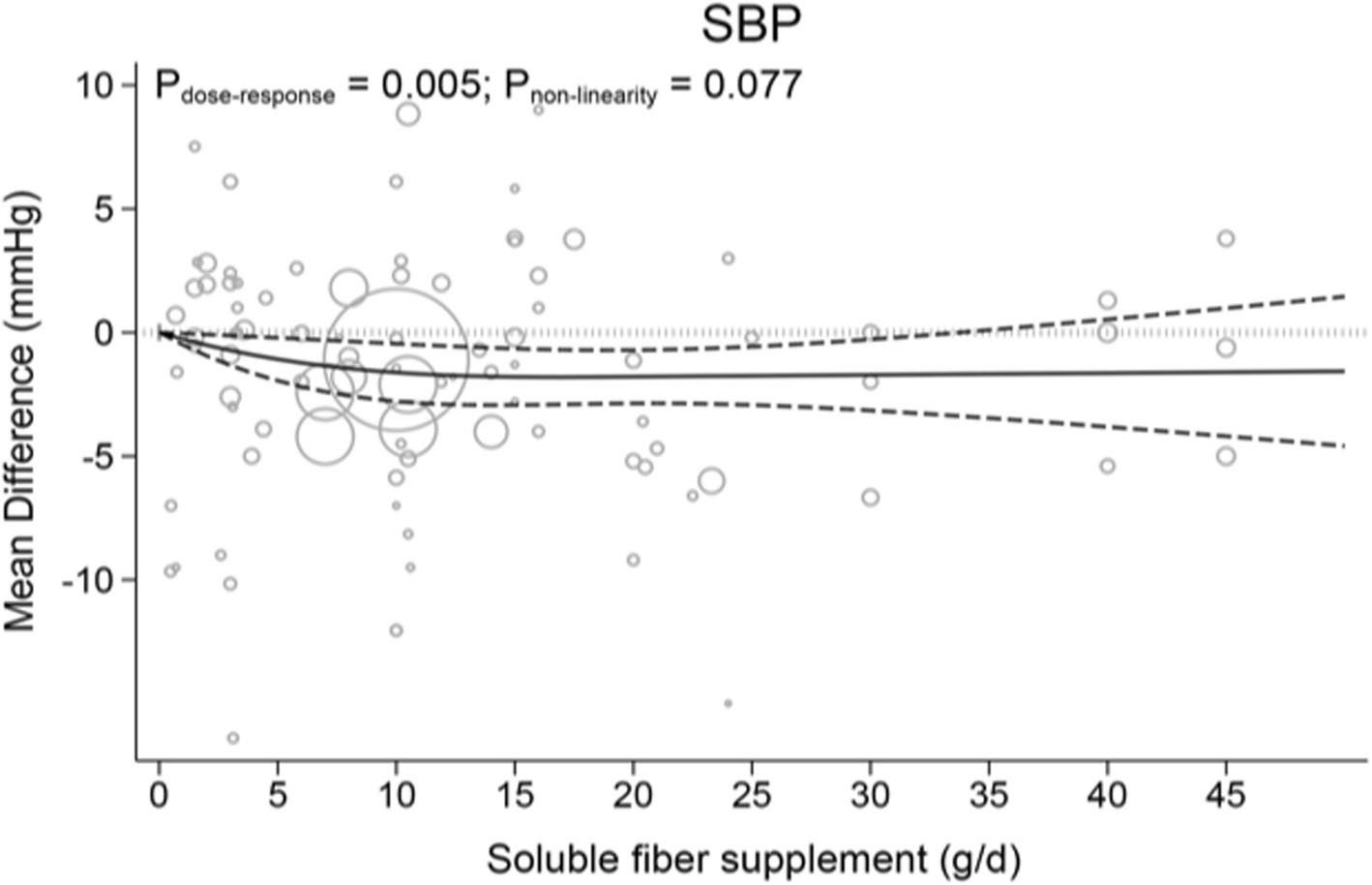
The effects of different doses of soluble fiber supplementation on SBP form the nonlinear dose response meta-analysis

Dose-dependent effect of soluble fiber supplementation on SBP are revealed in Fig. 2 and Table 2. Levels of SBP decreased proportionally with the increase in soluble fiber supplementation up to 20 g/d (MD_20g/d_: -1.79, 95%CI: -2.86, -0.71), and then appeared to plateau with a slight upward curve (*P*_nonlinearity_ = 0.077, *P*_dose-response_ = 0.005). There was no significant reduction in SBP after consuming ≥ 35 g/d of soluble fiber supplementation (MD_35g/d_: -1.68, 95%CI: -3.46, 0.10).

**Table 2:** The effects of different doses of soluble fiber supplements on blood pressure form the nonlinear dose–response meta-analysis (mean difference and 95% confidence interval)

Soluble fiber supplements (g/d)	0 (Ref)	5	8	10	15	20	25	30	35	40	45
SBP	0	-1.08 (-1.95, -0.21)	-1.46 (-2.56, -0.36)	-1.62 (-2.79, -0.45)	-1.79 (-2.94, -0.65)	-1.79 (-2.86, -0.71)	-1.75 (-2.93, -0.57)	-1.71 (-3.15, -0.28)	-1.68 (-3.46, 0.10)	-1.64 (-3.81, 0.53)	-1.61 (-4.19, 0.98)
DBP	0	-0.51 (-1.12, 0.09)	-0.72 (-1.49, 0.05)	-0.82 (-1.64, -0.01)	-0.99 (-1.80, -0.19)	-1.10 (-1.84, -0.36)	-1.20 (-1.96, -0.43)	-1.29 (-2.19, -0.40)	-1.39 (-2.48, -0.30)	-1.48 (-2.80, -0.16)	-1.58 (-3.15, -0.01)

**Fig. 3:**
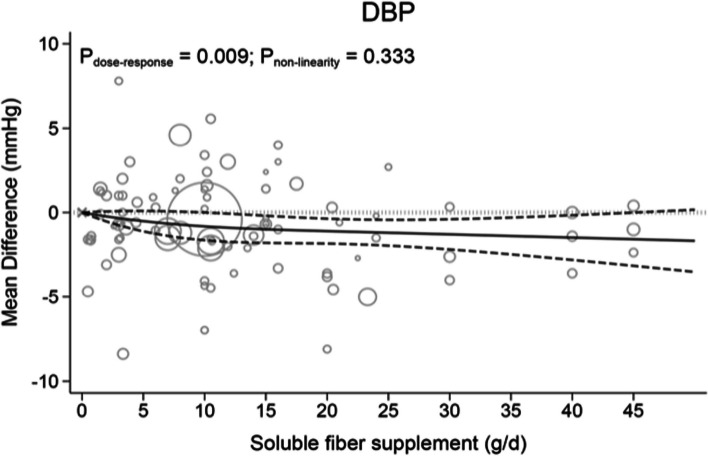
The effects of different doses of soluble fiber supplementation on DBP form the nonlinear dose response meta-analysis

Figure 3 and Table 2 demonstrate the dose-dependent effect of soluble fiber supplementation on DBP. DBP levels slightly and linearly decreased (*P*_nonlinearity_ = 0.333, *P*_dose-response_ = 0.009) up to a 45 g/d soluble fiber supplementation (MD_45g/d_: -1.58, 95%CI: -3.15, -0.01).

#### Sensitivity analysis and publication bias

**Fig. 4:**
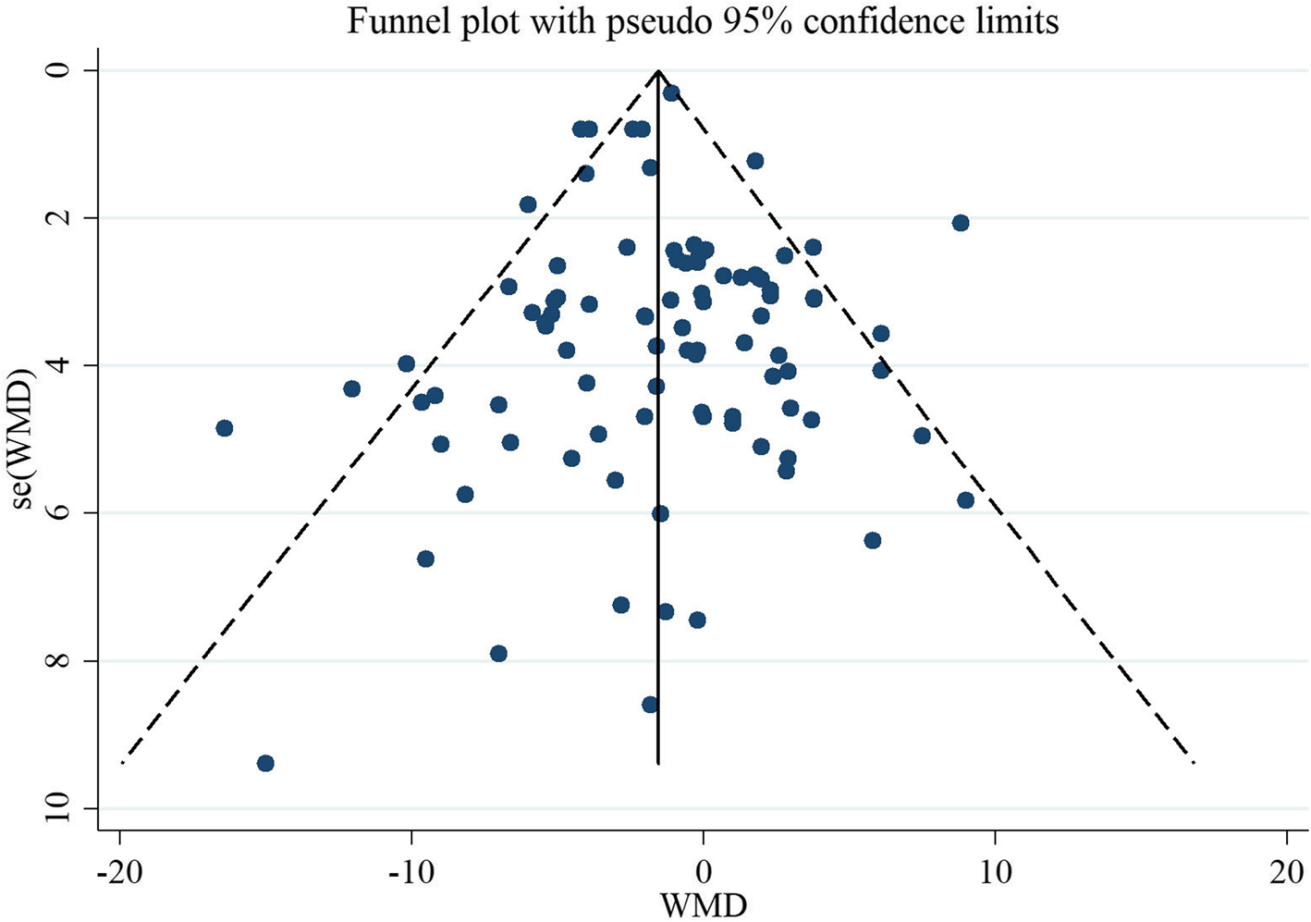
Funnel plot of studies evaluate SBP

**Fig. 5:**
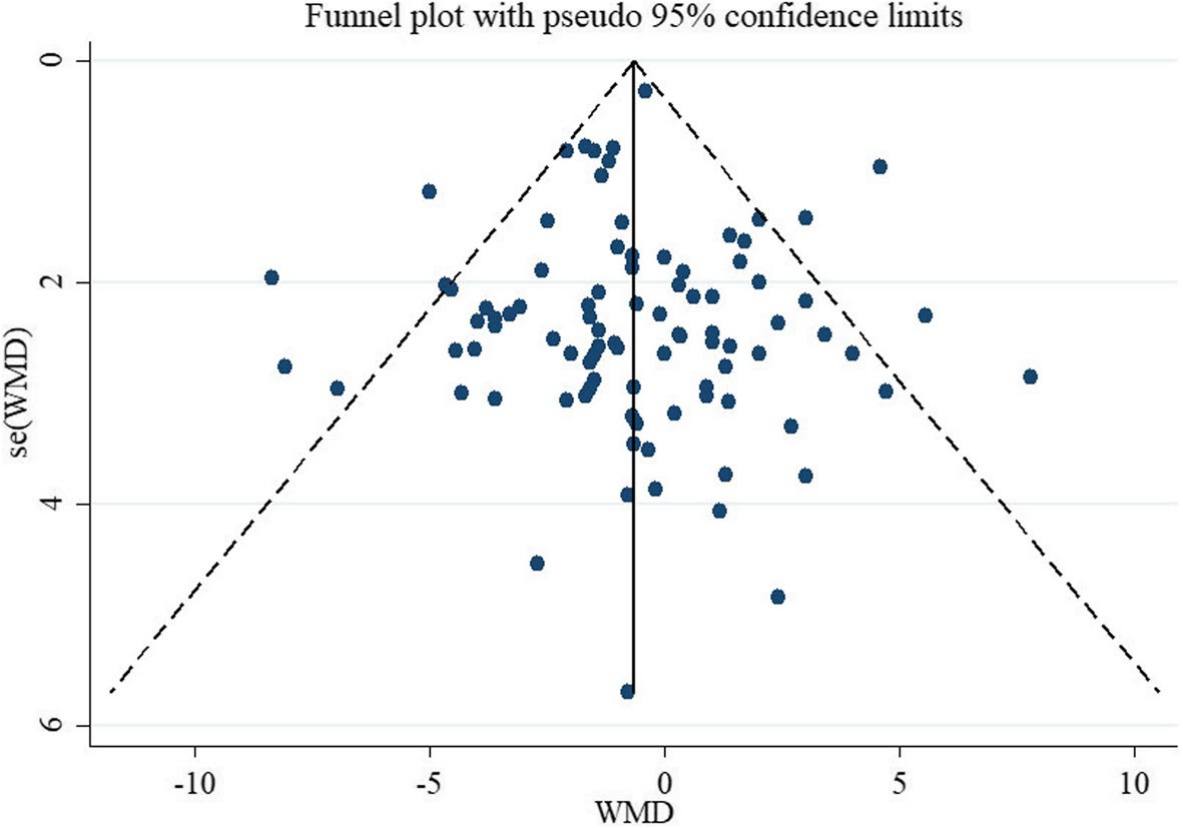
Funnel plot of studies evaluate DBP

To detect the impact of each study on the pooled effect size, each trial was excluded step-by-step from the pooled analysis, and their individuality was accounted for. We found no significant effects of any individual study on the pooled effect size. The evaluation of publication bias by visual inspection of the funnel plot illustrated a symmetry in SBP and DBP plots (Figs. 4 and 5). Also, Egger's test and Begg’s test revealed no evidence of publication bias for studies examining the effect of soluble fiber supplementation on SBP (0.875 Egger's test and 0.179 Begg’s test) and DBP (0.784 Egger's test and 0.463 Begg’s test).

#### Risk of bias and certainty of evidence

## Discussion

The present dose–response systematic review and meta-analysis summarized data from eighty-three RCTs, including 5867 participants, examining the effect of soluble fiber supplementation on blood pressure, and demonstrated significant reductions in both SBP and DBP following supplementation in a dose–response manner. In this study, fermentable soluble fibers induced greater reductions in both SBP and DBP compared to non-fermentable fibers. Also, the antihypertensive effect of soluble fibers was more pronounced in patients with hypertension and in guar gum and inulin subsets compared to other soluble fiber types.

To our knowledge, this study is the first to explore the blood pressure-lowering effects of all soluble fibers with comprehensive subgroup analyses based on soluble fiber type. Previous meta-analyses, which their findings regarding the beneficial effect of dietary fibers on blood pressure control were consistent with our study, were mainly done on the effects of all fiber types, irrespective of solubility, on blood pressure levels [8, 9] with the exception of one study which only considered viscous soluble fibers [10]. Streppel et al. initially performed a meta-analysis on the effects of all dietary fiber types including on blood pressure control. They found significant changes of DBP, but not SBP, following supplementation, which was larger for soluble than insoluble fibers [8]. Evans et al. found limited evidence on the impact of total fiber intake on SBP and DBP, but significant reductions of both SBP and DBP following higher doses of beta-glucan were demonstrated [27]. Since, studies in which more than 50% of the population were diagnosed with hypertension or those included participants with any abnormal health condition were excluded, the findings of this study cannot be extrapolated to general population including hypertensive patients. Khan et al., in a review study of viscous soluble fiber and blood pressure control, reported overall lowering effect of five fiber types on SBP and DBP, with a median dose of 8.7 g/day over a median follow-up of 7-weeks [10]. Consistent with our findings, they found greater reductions in SBP and DBP in patients with hypertension compared to normotensive individuals.

Blood pressure control is a cornerstone of approaches to reduce cardiovascular risk, as only 4 mmHg decrease in DBP is associated with a cardiovascular risk reduction of about 51% and a 10-mmHg reduction in SBP reduced the risk of major cardiovascular disease events by 20% and all-cause mortality by 13% [28, 29]. Although, level of SBP and DBP reduction found in our study might not be clinically important [30], increasing soluble fiber intake as a safe dietary intervention which is accessible for general population including those with hypertension might be considered a beneficial therapeutic strategy for blood pressure control and cardiometabolic risk reduction.

The underlying antihypertensive mechanisms of soluble fibers have not been clearly understood. Recent evidence supports the role of gut microbiota-derived metabolites such as short-chain fatty acids (SCFA) and trimethylamine N-oxide on blood pressure regulation, and introduces gut microbiota as a new target for managing hypertension. Most types of dietary fibers beneficially affect gut microbial composition inducing significant bifidogenic effect and increased levels of SCFAs. SCFAs and their receptors were associated with blood pressure regulation in animal studies and several mechanisms were suggested in this regard including regulation of neurotransmitter signaling, vasodilation and modulation of immune system and inflammatory responses [31]. Insulin resistance and compensatory hyperinsulinemia might be important contributors to hypertension through several mechanism including loss of insulin-induced vasodilation, activation of sympathetic nervous system and increased renal sodium reabsorption [32]. Soluble fibers may affect blood pressure by improving insulin sensitivity. Fermentable soluble fibers affect gut microbial composition and SCFA production, leading to increased levels of gut peptides such as glucagon-like peptide-1 (GLP-1) and peptide YY (PYY), which improve insulin secretion and delay gastric emptying [5]. This may explain the larger reduction in both SBP and DBP induced by fermentable soluble fibers in subgroup analyses. Also, soluble fibers, specifically viscous soluble fibers, delay the absorption of nutrients by increasing the viscosity of digest in the gastrointestinal tract. The resultant modulation of postprandial glucose response leads to improvement of insulin resistance [33]. The beneficial effect of soluble fibers on weight reduction is another potential mechanism to decrease blood pressure levels, as evidence exist on improved blood pressure control following weigh loss [34, 35]. This may account for the greater DBP reductions seen in our study in a subgroup of individuals with obesity. Another proposed mechanism is the lowering effect of soluble fibers on blood cholesterol, which may improve endothelium-mediated vasodilation and blood pressure control [36].

Moderate significant between-study heterogeneity was found in the analysis of both SBP and DBP. We performed several pre-specified subgroup analyses to detect sources of heterogeneity and found that participants’ health status and age, study duration and the fermentability of soluble fiber might influence the magnitude of effect; however, residual heterogeneity within some subgroups can not be excluded.

The present dose–response systematic review and meta-analysis is the first to explore the blood pressure-lowering effects of all soluble fibers with comprehensive subgroup analyses based on soluble fiber type. All relevant studies were included, regardless of study duration and participants’ baseline health condition (healthy and unhealthy individuals) which make our findings highly generalizable. However, several limitations of the study should be noted. First, the vehicle by which soluble fiber supplementation administered (supplements or food), which could have affected the efficacy of the supplementation, was not considered in subgroup analysis in our study. Second, we included studies with different target populations, including those with abnormal health conditions or receiving medication, which could have confounded our results. Third, since, we did not include studies on children or adolescents, our findings cannot be extrapolated to these age groups. Fourth, despite comprehensive subgroup analyses some sources of heterogeneity could have been remained and affected our estimates such as route for fiber administration (supplement vs. food), the method by which blood pressure was measured and high variability in the duration of trials (3 to 52 weeks) and supplementation dosage (0.477 to 45 g/day).

## Conclusion

Overall, the results of this systematic review and meta-analysis suggest that soluble fiber supplementation, in particular fermentable soluble fibers, beneficially affect SBP and DBP levels in a dose–response manner. This beneficial effect may be enhanced in individuals with hypertension. Since, intake of soluble fiber in western countries is below the recommended levels, increasing fiber intake in such populations may contribute to the prevention of hypertension and CVD risk reduction, as well as help to manage hypertension. More research is needed in normal-weigh individuals or with an attempt to adjust the changes in body weight, to better differentiate the contribution of soluble fibers, independently from weight loss, in blood pressure control. Also, future studies should consider the vehicle by which dietary fibers were administered, as this may impact the efficacy of the intervention.

### Supplementary Information

**Additional file 1: Table S1.** Search strategies including the key terms and the queries for each database. **Table S2.** Reason for exclusion of retrieved articles. **Table S3.** Characteristics of eligible studies examining the effect of soluble fiber supplementation on blood pressure parameters. **Table S4.** Sensitivity analyses of the use of correlation coefficients of 0.25 and 0.75. **Table S5.** Result of subgroup analysis of included studies in meta-analysis. **Table S6.** Risk of bias assessment in randomised controlled trials. **Table S7.** The GRADE evidence quality for each outcome.

## Data Availability

No additional data available.
