# Supplementary material for: Effect of soluble fiber on blood pressure in adults: a systematic review and dose–response meta-analysis of randomized controlled trials
Source: Nutr J. 2023 Oct 13;22:51. doi: 10.1186/s12937-023-00879-0 (PMC10571351; doi:10.1186/s12937-023-00879-0)
Supplement: Supplementary file 1 — Additional file 1: Table S1. Search strategies including the key terms and the queries for each database. Table S2. Reason for exclusion of retrieved articles. Table S3. Characteristics of eligible studies examining the effect of soluble fiber supplementation on blood pressure parameters. Table S4. Sensitivity analyses of the use of correlation coefficients of 0.25 and 0.75. Table S5. Result of subgroup analysis of included studies in meta-analysis. Table S6. Risk of bias assessment in randomised controlled trials. Table S7. The GRADE evidence quality for each outcome. [file 12937_2023_879_MOESM1_ESM.docx]

**Effect of soluble fiber on blood pressure in adults: A systematic review and dose-response Meta-analysis of randomized controlled trials**

**Supplementary material including: 5** **Tables**

Abed Ghavami, Sara Banpouri, Rahele Ziaei, Sepide Talebi, Mahdi Vajdi, Elyas Nattagh‐Eshtivani, Hanieh Barghchi, Hamed Mohammadi, Gholamreza Askari

| **Table S1.** Search strategies including the key terms and the queries for each database | |
| --- | --- |
| **Database** | **Key terms and the queries** |
| **PubMed** | #1 "Dietary Fiber"[MeSH Terms] OR "plant mucilage"[MeSH Terms] OR "glucan*"[MeSH Terms] OR "beta glucan*"[MeSH Terms] OR "plant mucilage"[MeSH Terms] OR "alginate*"[MeSH Terms] OR "gum Arabic"[MeSH Terms] OR "resistant starch"[MeSH Terms] OR "psyllium"[MeSH Terms] OR "inulin"[MeSH Terms] OR "Dietary Fiber"[MeSH Terms] OR "Dietary Fiber"[Title/Abstract] OR "soluble dietary fiber "[Title/Abstract] OR "soluble fibre"[Title/Abstract] OR "viscous fibre"[Title/Abstract] OR "inulin"[Title/Abstract] OR "psyllium"[Title/Abstract] OR "resistant starch"[Title/Abstract] OR "resistant maltodextrin"[Title/Abstract] OR "wheat dextrin"[Title/Abstract] OR "polydextrose"[Title/Abstract] OR "guar gum"[Title/Abstract] OR "plant gum"[Title/Abstract] OR "gum"[Title/Abstract] OR "gellan gum"[Title/Abstract] OR "gum Arabic"[Title/Abstract] OR "acacia gum"[Title/Abstract] OR "alginate*"[Title/Abstract] OR "plant mucilage"[Title/Abstract] OR "beta-glucan"[Title/Abstract] OR "beta glucan*"[Title/Abstract] OR "glucan*"[Title/Abstract] OR "prebiotic"[Title/Abstract] OR "fermentable fiber"[Title/Abstract] OR "prebiotic fiber"[Title/Abstract] OR "galactomannan"[Title/Abstract] OR "konjac"[Title/Abstract] OR "soluble corn fiber"[Title/Abstract] OR "fructooligosaccharide"[Title/Abstract] OR "oligofructose"[Title/Abstract] OR "galactooligosaccharide"[Title/Abstract] OR "pectin"[Title/Abstract] OR "plant mucilage"[Title/Abstract] OR "oat"[Title/Abstract] OR "barley"[Title/Abstract] OR "soluble dietary fiber"[Title/Abstract] OR "Dietary Fiber"[Title/Abstract].  #2 "Randomized Controlled Trial"[Publication Type]) OR ("Randomized Controlled Trial"[Title/Abstract])) OR (“Controlled Clinical Trial”[Title/Abstract])) OR (“Controlled Clinical Trial”[Publication Type])) OR (“clinical trial”[Title/Abstract])) OR (“randomized”[Title/Abstract])) OR (“placebo”[Title/Abstract])) OR ("intervention studies"[Title/Abstract])) OR ("intervention"[Title/Abstract])) OR ("controlled trial"[Title/Abstract])) OR ("random"[Title/Abstract])) OR ("randomly"[Title/Abstract])) OR ("blind"[Title/Abstract])) OR (“RCT”[Title/Abstract])) OR (“trial “[Title/Abstract]).  #3 #1 AND #2 |
| **Web of Science (ISI)** | #1 TOPIC: (" Dietary Fiber ”) OR TOPIC: (" soluble dietary fiber ") OR TOPIC: (" soluble fibre ") OR TOPIC: (" viscous fibre ") OR TOPIC: (" inulin ") OR TOPIC: (" psyllium ") OR TOPIC: (" psyllium ") OR TOPIC: (" resistant starch ") OR TOPIC: (" resistant maltodextrin ") OR TOPIC: (" wheat dextrin ") OR TOPIC: (" polydextrose ") OR TOPIC: (" guar gum ") OR TOPIC: (" plant gum ") OR TOPIC: (" gum ") OR TOPIC: (" gellan gum ") OR TOPIC: (" gum Arabic ") OR TOPIC: (" acacia gum ") OR TOPIC: (" alginate ") OR TOPIC: (" plant mucilage ") OR TOPIC: (" beta-glucan ") OR TOPIC: (" glucan ") OR TOPIC: (" prebiotic ") OR TOPIC: (" fermentable fiber ") OR TOPIC: (" galactomannan ") OR TOPIC: (" konjac ") OR TOPIC: soluble corn fiber ") OR TOPIC: (" fructooligosaccharide ") OR TOPIC: (" oligofructose ") OR TOPIC: (" galactooligosaccharide ") OR TOPIC: (" oat ") OR TOPIC: (" barley ")  #2 TOPIC: ("randomized controlled trial") OR TOPIC: ("clinical trial") OR TOPIC: ("randomized") OR TOPIC: ("placebo") OR TOPIC: ("intervention studies") OR TOPIC: ("intervention") OR TOPIC: ("controlled trial") OR TOPIC: ("random") OR TOPIC: ("randomly") OR TOPIC: ("blind") OR TOPIC: ("RCT") OR TOPIC: ("trial")  #3 #1 AND #2 |
| **Scopus** | #1 TITLE-ABS-KEY ( " Dietary Fiber " ) OR TITLE-ABS-KEY ( " soluble dietary fiber " ) OR TITLE-ABS-KEY ( " soluble fibre " ) OR TITLE-ABS-KEY ( " viscous fibre " ) OR TITLE-ABS-KEY ( " inulin " ) OR TITLE-ABS-KEY ( " psyllium " ) OR TITLE-ABS-KEY ( " resistant starch " ) OR TITLE-ABS-KEY ( " resistant maltodextrin " ) OR TITLE-ABS-KEY ( " wheat dextrin " ) OR TITLE-ABS-KEY ( " polydextrose " ) OR TITLE-ABS-KEY ( " guar gum " ) OR TITLE-ABS-KEY ( " plant gum " ) OR TITLE-ABS-KEY ( " gum " ) OR TITLE-ABS-KEY ( " gellan gum " ) OR TITLE-ABS-KEY ( " gum Arabic " ) OR TITLE-ABS-KEY ( " acacia gum " ) OR TITLE-ABS-KEY ( " alginate " ) OR TITLE-ABS-KEY ( " plant mucilage " ) OR TITLE-ABS-KEY ( " beta-glucan " ) OR TITLE-ABS-KEY ( " fermentable fiber " ) OR TITLE-ABS-KEY ( " galactomannan " ) OR TITLE-ABS-KEY ( " soluble corn fiber " ) OR TITLE-ABS-KEY ( " fructooligosaccharide " ) OR TITLE-ABS-KEY (“oligofructose " ) OR TITLE-ABS-KEY ( " konjac " ) OR TITLE-ABS-KEY ( " galactooligosaccharide " ) OR TITLE-ABS-KEY ( " oat " ) OR TITLE-ABS-KEY ( " barley " ).  #2 TITLE-ABS-KEY ( "randomized controlled trial" ) OR TITLE-ABS-KEY ( "clinical trial" ) OR TITLE-ABS-KEY ( "randomized" ) OR TITLE-ABS-KEY ( "placebo" ) OR TITLE-ABS-KEY ( "intervention studies" ) OR TITLE-ABS-KEY ( "intervention" ) OR TITLE-ABS-KEY ( "controlled trial" ) OR TITLE-ABS-KEY ( "random" ) OR TITLE-ABS-KEY ( "randomly" ) OR TITLE-ABS-KEY ( "blind" ) OR TITLE-ABS-KEY ( "RCT") OR TITLE-ABS-KEY ( "trial " ).  #3 #1 AND #2 |

**Table S2**: Reason for exclusion of retrieved articles

| **References** | **Reason for exclusion** |
| --- | --- |
| (1) Nishimura, A., et al., *Effect of functional barley BARLEYmax (Tantangara) on intestinal regulation: A double-blind, randomized, placebo-controlled parallel group comparison clinical study.* Japanese Pharmacology and Therapeutics, 2017. **45**(6): p. 1047-1055  (2) Jakeman, S., et al., *Soluble corn fiber increases bone-calcium retention in postmenopausal women in a dose-dependent manner.* Journal of Bone and Mineral Research, 2015. **30**  (3) Wolever, T.M.S., et al., *Effect of serving size and addition of sugar on the glycemic response elicited by oatmeal: A randomized, cross-over study.* Clinical Nutrition ESPEN, 2016. **16**: p. 48-54  (4) Zabriskie, H.A., et al., *Yeast beta-glucan supplementation downregulates markers of systemic inflammation after heated treadmill exercise.* Nutrients, 2020. **12**(4) | Insufficient data |
| (5) Kamal, E., et al., *Dietary Fibers (Gum Arabic) Supplementation Modulates Hepatic and Renal Profile Among Rheumatoid Arthritis Patients, Phase II Trial.* Front Nutr, 2021. **8**: p. 552049. | No control group |
| (6) Benítez-Páez, A., et al., *Sex, Food, and the Gut Microbiota: Disparate Response to Caloric Restriction Diet with Fiber Supplementation in Women and Men.* Molecular nutrition & food research, 2021. **65**(8): p. e2000996. | Article with similar exposure and outcome variable (duplicates) |
| (7) Riikonen, S., et al., *Oral guar gum, a gel-forming dietary fiber relieves pruritus in intrahepatic cholestasis of pregnancy.* Acta Obstetricia et Gynecologica Scandinavica, 2000. **79**(4): p. 260-264 | Pregnant women |

| **Table S3:** Characteristics of eligible studies examining the effect of soluble fiber supplementation on blood pressure parameters. | | | | | | | | | | | | | | | |
| --- | --- | --- | --- | --- | --- | --- | --- | --- | --- | --- | --- | --- | --- | --- | --- |
| **Study (year)**  **ref** | **Participants Characteristic** | | | | **Study Characteristic** | | | | **Intervention/Control Characteristic** | | | | | | |
|  | **Population** | **Sex (M/F)** | **Mean BMI (kg/m^2^)** | **Mean Age**  **(y)** | **Location** | **RCT design** | **Duration**  **(week)** | **Blinding** | **Fiber** | | | **Dose**  **(g/d)** | **Form of Administration** | **Comparator** | **Back**  **ground Diet** |
|  |  |  |  |  |  |  |  |  | **type** | **Viscosity** | **Ferment ability** |  |  |  |  |
| Momenizadeh (2014) (1) | HCL | Both | 28.965 | 51.12 | Iran | P | 6 | No | Beta-glucan | Yes | Yes | 30 | Premixed Food (bread) | Wheat fiber | Hypocaloric diet |
| Jarrar (2021) (2) | Adults at Risk of MetS | Both | 33 | 26.95 | UAE | P | 12 | S | Gum Arabic | Yes | Yes | 20 | Sachet | Pectin | Usual diet |
| Reimer (2020) (3) | T2DM | Both | 39.95 | 54.8 | Canada | P | 52 | D | PGX | Yes | Yes | 17.5 | Meal replacements plus supplement packets | Rice flour | Low calorie diet |
| Zhang (2020) (4) | SCZ with DLP | Both | 25.77 | 32 | China | P | 4 | D | Glucomannan | Yes | Yes | 6 | Beverage | RMD | Usual diet |
| Hiel (2020) (5) | Obese | Both | 36.35 | 41.5 | Belgium | P | 12 | S | Native inulin | No | Yes | 16 | Sachet | Maltodextrin | Hypocaloric diet |
| Cicero (2020) (6) | Mild HCL | Both | NR | 52.3 | Italy | C | 4 | D | Beta‐glucan | Yes | Yes | 3 | Sachet | Without beta‐glucan | Mediterranean diet |
| Kitagawa (2019) (7) | Healthy adults | Both | 26.5 | 47.1 | Japan | P | 12 | D | RMD | No | Yes | 15 | Beverage | Placebo beverage | Usual diet |
| Peterson (2018) (8) | Adults with prediabetes | Both | 35.6 | 54.5 | UK | P | 12 | D | RS2 | No | Yes | 45 | Yogurts | Amylopectin | Usual diet |
| Vaghef-Mehrabany (2019) (9) | Obese women with depression | F | 34.03 | 35 | Iran | P | 8 | D | Inulin | No | Yes | 10 | Sachet | Maltodextrin | Calorie-restricted diet |
| Abbasalizad Farhangi (2019) (10) | T2DM | F | 31.75 | 49.05 | Iran | P | 8 | T | Resistant dextrin | Yes | Yes | 10 | Powder | Maltodextrin | Usual diet |
| Lundby Hess (2019) (11) | Overweight or obese | Both | 33.75 | 48.59 | Denmark | P | 12 | D | Inulin plus RMD | No | Yes | 20 | Milk | Placebo supplementation | Restricted diet |
| Abbasalizad Farhangi (2016) (12) | T2DM | F | 30.7 | 48.34 | Iran | P | 8 | D | Chicory inulin enriched with oligofructose | No | Yes | 10 | Packages | Maltodextrin | Usual diet |
| Babiker (2018) (13) | T2DM | Both | 28.81 | 50.09 | Sudan | P | 12 | D | Gum Arabic | Yes | Yes | 30 | Powder | Pectin | Usual diet |
| Gulati (2017) (14) | Mild HCL | Both | 25.55 | 31.2 | India | P | 4 | No | Oats | Yes | Yes | 3 | Porridge and upma | No oat | Usual diet |
| Machado (2018) (15) | Overweight adults | Both | 30.44 | 31.34 | Brazil | P | 6 | D | FOS | Yes | Yes | 25 | Drink | Drink without yacon flour | Energy-restricted diets |
| Tabesh (2014) (16) | HCL | Both | 28.9 | 51.1 | Iran | P | 4 | No | Beta-glucan | Yes | Yes | 6 | Bread | Wheat fiber | Hypocaloric diet |
| Sartore (2009) (17) | T2DM | Both | 30.28 | 60.5 | Italy | P | 8 | No | Psyllium | Yes | No | 10.5 | Sachet | No psyllium | Controlled diet |
| Pal (2011) (18) | Overweight and obese | Both | 33.85 | 43.05 | Australia | P | 12 | S | Psyllium | Yes | No | 21 | Sachet | Breadcrumbs | Usual diet |
| de Souza (2019) (19) | MetS | Both | 34.7 | 47.6 | Brazil | P | 6 | No | Oat bran | Yes | Yes | 40 | NR | Without oat bran | Low-calorie diet |
| Abutair (2018) (20) | T2DM | Both | NR | 47.27 | Palestine | P | 8 | No | Psyllium | Yes | No | 10.5 | Soluble fiber supplementation | No dietary intervention | Usual diet |
| Solà (2010) (21) | HCL | Both | 27.13 | 54.91 | Spain | P | 8 | D | Soluble fibre | Yes | Yes | 14 | Sachets | Microcrystalline-cellulose | Low saturated fat diet |
| Mosikanon (2016) (22) | Overweight and Obese | Both | 27.7 | 41.27 | Thailand | P | 6 | D | β–glucan | Yes | Yes | 0.47 | Capsule | Rice flour | Usual diet |
| Dehghan (2016) (23) | T2DM | F | 30.7 | 48.34 | Iran | P | 8 | D | Oligofructose enriched inulin | No | Yes | 10 | NR | Maltodextrin | Usual diet |
| Chang (2013) (24) | Obese | Both | 29.36 | 38.55 | Taiwan | P | 12 | D | Beta glucan | Yes | Yes | 1.5 | Cereal | Without beta-glucan | Usual diet |
| Lee (2017) (25) | Healthy adults | Both | 23.35 | 32.15 | Korea | P | 8 | D | β-1,3-glucan | Yes | Yes | 0.7 | Capsule | Cellulose | Usual diet |
| Jensen (2012) (26) | Obese | Both | 34.2 | 42.9 | Denmark | P | 12 | D | Alginate | Yes | Yes | 45 | Beverage | Maltodextrin | Energy-restricted diet |
| Davy (2002) (27) | High-Normal BP | M | 29.4 | 59 | USA | P | 12 | No | Beta-glucan | Yes | Yes | 14 | Cereals | Wheat | Usual diet |
| Wood (2007) (28) | Overweight and obese | M | 29.7 | 38.8 | USA | P | 12 | D | Konjac-mannan | Yes | Yes | 3 | Capsules | Maltodextrin | Carbohydrate-restricted diet |
| Tessari (2017) (29) | T2DM | Both | 27.85 | 68.3 | Italy | P | 26 | No | Beta Glucan | Yes | Yes | 7.6 | Bread | No beta Glucan | Usual diet |
| Saltzman (2001) (30) | Healthy weight-stable | Both | 26.4 | 44.6 | USA | P | 6 | No | Oats | Yes | Yes | 45 | Combination of cereal , beverage , breads and casseroles | No oat | Hypocaloric diet |
| Dall’Alba (2013) (31) | T2DM | Both | 29.75 | 62.05 | Brazil | P | 6 | No | PHGG | Yes | Yes | 10 | Sachet | No PHGG | Usual diet |
| Anderson (1988) (32) | HCL | M | 24.25 | 46.75 | USA | P | 8 | D | PHM | Yes | No | 10.2 | Sachet | Cellulose | Usual diet |
| Bell (1989) (33) | Mild to Moderate HCL | Both | NR | 47.35 | USA | P | 8 | D | PHM | Yes | No | 10.2 | Sachet | Microcrystalline cellulose | Prudent, low-fat diet |
| Levin (1990) (34) | Mild to Moderate HCL | Both | 25.55 | 50.75 | USA | P | 16 | D | PHM | Yes | No | 10.2 | Sachet | Cellulose | AHA Step I Diet |
| Liatis (2009) (35) | T2DM | Both | 28.31 | 63.36 | Greece | P | 3 | D | Betaglucan | Yes | Yes | 3 | Bread | No betaglucan | Usual diet |
| Makkonen (1993) (36) | Menopausal women | F | 30.9 | 52.95 | Finland | P | 26 | D | Guar gum | Yes | Yes | 15 | Granule | Wheat flour | Usual diet |
| Maeda (2005) (37) | Obese with IGT and T2DM | Both | 28.8 | 58.6 | Japan | P | 12 | No | Agar (kanten) | Yes | Yes | 4.5 | Gel | No agar | Balanced calorie-reduced diet |
| J. Pins (2002) (38) | HTN | Both | 30.9 | 47.55 | USA | P | 12 | No | Whole oat | Yes | Yes | 23.3 | Cereals | Wheat | Usual diet |
| Park (2004) (39) | Healthy overweight | F | 27.25 | 42.95 | Korea | P | 3 | D | RS | Yes | Yes | 24 | Powder | CS | Usual diet |
| Azezli (2007) (40) | Hyperthyroid | Both | 24.05 | 39.1 | Turkey | P | 8 | S | KGM | Yes | Yes | 2.6 | Powder | No KGM | Usual diet |
| Gómez-Reyes (2010) (41) | IHD | Both | 31.43 | 65.18 | Mexico | P | 12 | D | Inulin and oligofructose | No | Yes | 3.35 | Bread | No inulin and oligofructose | Usual diet |
| Neyrinck (2021) (42) | Obese | NR | 35.5 | NR | Belgium | P | 12 | S | Native inulin | No | Yes | 16 | Powder | Maltodextrin | Hypocaloric diet |
| Aoe (2017) (43) | visceral fat obesity | Both | 27.55 | 50 | Japan | P | 12 | D | High-β-glucan barley | Yes | Yes | 4.4 | Sachet | β-glucan-free barley | Usual diet |
| Liao (2018) (44) | Mildly HCL | NR | 23.52 | 52.5 | Taiwan | P | 10 | D | Oat | Yes | Yes | 3.12 | Noodle | Wheat | Usual diet |
| Reimer (2013) (45) | Adults with Abdominal Obesity | Both | 26.95 | 42.5 | Canada | P | 14 | D | PGX | Yes | Yes | 15 | Sachet | Rice flour | Usual diet |
| Benítez-Páez (2021) (46) | Overweight | Both | 33.55 | NR | Spain | P | 12 | D | Inulin and RMD | No | Yes | 40 | Sachet | Maltodextrin | Caloric restriction diet |
| Vuksan (2000) (47) | IRS | Both | 28 | 55 | Canada | C | 3 | D | Glucomannan | Yes | Yes | 10.5 | Biscuits | Wheat bran | Usual diet |
| Zhang (2012) (48) | Mild to moderate HCL | Both | 25.5 | 53.2 | China | P | 6 | No | Oat | Yes | Yes | 3.6 | Meal | Wheat | Usual diet |
| Hokazono (2010) (49) | High Serum Uric Acid or Mild Hyperuricemia | Both | 25.1 | 43.45 | Japan | P | 12 | D | FBEP | Yes | Yes | 2 | Drink | NO FBEP | Usual diet |
| Nishimura (2015) (50) | Healthy adult | Both | 21.9 | 53.61 | Japan | P | 4 | D | inulin-type fructans | No | Yes | 10 | Drink | Barley tea | Usual diet |
| Cai (2018) (51) | T2DM | NR | 27.77 | 60.55 | China | P | 12 | D | Inulin and resistant dextrin | No | Yes | 20.5 | Powder | No Inulin and resistant dextrin | Usual diet |
| Tuomilehto (1988) (52) | Severe HCL | Both | NR | 56.9 | Finland | P | 50 | No | Guar gum | Yes | Yes | 22.5 | Granules | Wheat | Usual diet |
| Raimondi de Souza (2016) (53) | HCL | Both | 28.95 | 55.75 | Brazil | P | 12 | D | OB | Yes | Yes | 40 | Powder | Rice flour | Usual diet |
| NEAL (1990) (54) | HCL | Both | NR | NR | USA | P | 13 | No | Psyllium | Yes | No | 20.4 | Sachet | No Psyllium | Phase I AHA Diet |
| Cheang (2017) (55) | MetS | Both | 28.74 | 52.5 | Taiwan | C | 4 | D | Glucomannan | Yes | Yes | 2 | Noodles | Without glucomannan | Usual diet |
| Eshghi (2019) (56) | Overweight and Obese Adults | Both | 32.4 | 35 | Iran | C | 4 | D | RS | Yes | Yes | 13.5 | Supplements | Maltodextrin | Usual diet |
| Önning (1999) (57) | Moderate HCL | M | 27.1 | 62.55 | Sweden | P | 5 | D | Oat (ß-glucan) | Yes | Yes | 0.75 | Drink | Rice | Usual diet |
| Arvill (1995) (58) | Healthy | M | 28.7 | 47 | Sweden | C | 8 | D | KGM | Yes | Yes | 3.9 | Gelatin capsules | CS | Usual diet |
| Sakai (2019) (59) | T2DM | Both | 25.01 | 59.1 | Japan | C | 12 | D | Fucoidan | Yes | Yes | 1.62 | Beverage | No fucoidan | Usual diet |
| Uusitupa (1989) (60) | NIDDM | Both | 28.4 | 60.12 | Finland | P | 52 | D | Guar gum | Yes | Yes | 15 | Granules | Wheat flour | Usual diet |
| Upadhyaya (2016) (61) | MetS | Both | 32.8 | NR | USA | C | 24 | D | RS4 | Yes | Yes | NR | Flour | No RS4 | Usual diet |
| Jenkins (2002) (62) | HLP | Both | 25.6 | 60 | Canada | C | 4 | No | B-glucan or psyllium | No | Yes | 8 | Sachets | No B-glucan or psyllium | Usual diet |
| Vuksan (1999) (63) | T2DM | Both | NR | 60.5 | Canada | C | 3 | D | KJM | Yes | Yes | 10.6 | Biscuits | Wheat bran | Low-fat diet |
| Penn-Marshall (2010) (64) | At risk for T2DM | Both | 37.7 | 36.6 | USA | C | 6 | D | RS | Yes | Yes | 12.39 | Bread | No RS | Usual diet |
| Nichenametla (2014) (65) | MetS | Both | 30.9 | 51.7 | USA | C | 12 | D | RS4 | Yes | Yes | NR | Flour | No RS4 | Usual diet |
| Schwab (2006) A (66) | AGM | Both | 28.86 | 53 | Finland | P | 12 | D | SBP | Yes | Yes | 16 | Drink | No SBP | Usual diet |
| Schwab (2006) B (67) | AGM | Both | 28.86 | 53 | Finland | P | 12 | D | PDX | Yes | Yes | 16 | Drink | No PDX | Usual diet |
| Lupton (1994) A (68) | HCL | Both | NR | 48.2 | USA | P | 4 | No | Barley bran | Yes | Yes | 30 | Flour | Cellulose | Fat-modified diet |
| Lupton (1994) B (69) | HCL | Both | NR | 48.2 | USA | P | 4 | No | Barley | Yes | Yes | 3 | Oil extract | Cellulose | Fat-modified diet |
| Geliebter (2014) (70) | Overweight | Both | 32.8 | 33.9 | USA | P | 4 | No | Oat | Yes | Yes | 8 | Porridge | No oat | Usual diet |
| Cicero (2007) A (71) | HTN Overweight | Both | 26.63 | 57.83 | Italy | P | 24 | D | Psyllium | Yes | No | 10.5 | Powder | No psyllium | Usual diet |
| Cicero (2007) B (72) | HTN Overweight | Both | 26.63 | 57.83 | Italy | P | 24 | D | Guar gum | Yes | Yes | 10.5 | Powder | No guar gum | Usual diet |
| Bell (1990) A (73) | mild to moderate HCL | M | NR | 46 | USA | P | 6 | D | Pectin | Yes | Yes | 3 | Cereals | Corn flakes | Usual diet |
| Bell (1990) B (74) | mild to moderate HCL | M | NR | 45.5 | USA | P | 6 | D | Psyllium | Yes | No | 5.8 | Cereals | Corn flakes | Usual diet |
| Chen (2016) A (75) | T2DM | Both | 25.22 | 52.57 | China | P | 4 | D | NR | NR | NR | 10 | NR | No dietary fiber | ADA diet |
| Chen (2016) B (76) | T2DM | Both | 25.48 | 53.52 | China | P | 4 | D | NR | NR | NR | 20 | NR | No dietary fiber | ADA diet |
| Charlton (2012) A (77) | Mildly HCL overweight | Both | 27.51 | 50.84 | Australia | P | 6 | S | Oat b-glucan (low) | Yes | Yes | 1.5 | RTE oat flakes and puffed rice and wheat bars | Minimal b-glucan | Low-fat diet |
| Charlton (2012) B (78) | Mildly HCL overweight | Both | 27.24 | 51.09 | Australia | P | 6 | S | Oat b-glucan (high) | Yes | Yes | 3.2 | RTE oat flakes and puffed rice and wheat bars | Minimal b-glucan | Low-fat diet |
| Roshanravan (2017) (79) | T2DM | Both | 30.61 | 51.6 | Iran | P | 6 | D | Inulin | No | Yes | 10 | Powder | Starch | Usual diet |
| Cicero (2009) A (80) | MetS | Both | 28.65 | 57.8 | Italy | P | 24 | No | Psyllium | Yes | No | 7 | Powder | No psyllium | AHA Step 2 diet |
| Cicero (2009) B (81) | MetS | Both | 28.55 | 57.55 | Italy | P | 24 | No | PHGG | Yes | Yes | 7 | Powder | No guar gum | AHA Step 2 diet |
| Ghalandari (2018) (82) | T2DM | Both | 29.5 | 52.9 | Iran | P | 8 | S | Psyllium | Yes | No | 3.1 | Powder | CS | Calorie-restricted diets |
| He (2004) (83) | HTN | Both | 28.9 | 47.9 | USA | P | 12 | D | Oat bran | Yes | Yes | 8 | Muffin and cereals | Wheat | Usual diet |
| Ibrugger (2013) A (84) | Healthy Adults | Both | 22.8 | 22.9 | Denmark | C | 3 | S | Oat | Yes | Yes | 3.3 | Beverage and yogurt | NR | Usual diet |
| Ibrugger (2013) B (85) | Healthy Adults | Both | 22.8 | 22.9 | Denmark | C | 3 | S | Barley | Yes | Yes | 3.3 | Beverage and yogurt | NR | Usual diet |
| Ibrugger (2013) C (86) | Healthy Adults | Both | 22.8 | 22.9 | Denmark | C | 3 | S | Barley mutant | Yes | Yes | 3.3 | Beverage and yogurt | NR | Usual diet |
| Tripkovic (2014) (87) | Overweight | M | 30.2 | 39.8 | UK | C | 4 | No | Inulin | No | Yes | 15 | Bread | Refined wheat grain | Usual diet |
| Burke (2001) (88) | Treated HTN | Both | NR | 56.55 | Australia | P | 8 | No | Psyllium | Yes | No | 15 | Powder | Maltodextrin | Usual diet |
| Johnstone (2020) (89) | Overweight | Both | 33.05 | 41 | UK | C | 7 | No | RS3 | Yes | Yes | 24 | Meals | No RS3 | Usual diet |
| Vuksan (2020) A (90) | T2DM | Both | 31 | 60 | Canada | C | 3 | No | KBB | Yes | Yes | 0.7 | Biscuits | Wheat bran | NCEP Step 2 |
| Vuksan (2020) B (91) | IGT/ MetS | Both | 28 | 55 | Canada | C | 3 | D | KJM | Yes | Yes | 0.5 | Biscuits | Wheat bran | NCEP Step 2 |
| Jenkins (1997) A (92) | HCL | Both | 24.7 | 57.5 | Canada | C | 4 | No | Psyllium | Yes | No | 11.9 | Cereal | Wheat bran | Metabolic diets (6% MUFA diet ) |
| Jenkins (1997) B (93) | HCL | Both | 25.8 | 58 | Canada | C | 4 | No | Psyllium | Yes | No | 11.9 | Cereal | Wheat bran | Metabolic diets (12% MUFA diet ) |
| Wong (2013) A (94) | Healthy older adults-Antihypertensive medication | Both | 26.4 | 67 | Australia | C | 12 | D | Wild green oat extract (Neuravena) | Yes | Yes | 1.5 | Capsule | Microcrystalline cellulose | Usual diet |
| Wong (2013) B (95) | Healthy older adults-cholesterol-lowering medication | Both | 26.4 | 67 | Australia | C | 12 | D | Wild green oat extract (Neuravena) | Yes | Yes | 1.5 | Capsule | Microcrystalline cellulose | Usual diet |

Abbreviation: AHA, American Heart Association; AGM, Abnormal glucose metabolism; BP, Blood pressure; C, cross-over; D, double; CS, Corn starch; DLP, Dyslipidemia; F, Female; FOS, Fructooligosaccharides; FBEP, Fermented barley extract P; HCL, Hypercholesterolemic; HTN, Hypertension; HLP, Hyperlipidemic; IGT, impaired glucose tolerance; IHD, Ischemic heart disease; IRS, Insulin Resistance Syndrome; KGM, Konjac glucomannan; KJM, Konjac-mannan; KBB, konjac-based blend; MetS, metabolic syndrome; M, Male; NCEP, National Cholesterol Education Program; NIDDM, Non-insulin-dependent diabetes mellitus; NR, not reported; OB, Oat bran; P, parallel; PGX, PolyGlycopleX; PHGG, Partially hydrolysed guar gum; PDX, Polydextrose; PHM, Psyllium Hydrophilic Mucilloid; RMD, Resistant maltodextrin; RS, resistant starch; S, Single; SBP, Sugar beet pectin; SCZ, Schizophrenia; T2DM, Type 2 diabetes mellitus; T, triple;

| **Table** **S4**: Sensitivity analyses of the use of correlation coefficients of 0.25 and 0.75 | | | |
| --- | --- | --- | --- |
| Outcome | MD (95% CI), P-value, I^2^, P-value _heterogeneity_ | | |
|  | Correlation coefficient used in the primary analysis | Correlation coefficient used in sensitivity analyses | |
|  | 0.5 | 0.25 | 0.75 |
| SBP | -1.36 (-2.13, -0.60), P<0.001, I^2^=47.1, P_het_<0.001 | -1.42 (-2.21, -0.63), P<0.001, I^2^=31.3, P_het_=0.003 | -1.33 (-2.03, -0.63), P<0.001, I^2^=67.8, P_het_<0.001 |
| DBP | -0.72 (-1.26, -0.18), P=0.009, I^2^=45.4, P_het_<0.001 | -0.73 (-1.27, -0.19), P=0.008, I^2^=26.0, P_het_=0.014 | -0.71 (-1.22, -0.19), P=0.007, I^2^=68.4, P_het_<0.001 |

**Abbreviations:** MD; Mean Difference, CI; Confidence Interval, SBP; Systolic Blood Pressure, DBP; Diastolic Blood Pressure

**Table S5.** Result of subgroup analysis of included studies in meta-analysis.

| **Sub-grouped by** | **No. of trials** | **Effect size^1^** | **95% CI, Pvalue** | **I^2^ (%)** | **P for heterogeneity** | | **P for between**  **subgroup heterogeneity** |
| --- | --- | --- | --- | --- | --- | --- | --- |
| **SBP (All trials)** | **94** | **-1.36** | **(-2.13, -0.60), <0.001** | **47.1** | **<0.001** |  | |
| Duration |  |  |  |  |  | | **0.039** |
| < 8 weeks | 40 | -0.71 | (-1.82, 0.39), 0.206 | 7.6 | 0.328 | |  |
| ≥ 8 weeks | 54 | -1.61 | (-2.59, -0.62), 0.001 | 59.0 | <0.001 | |  |
| Dose |  |  |  |  |  | | **0.343** |
| ≤ 10 gr/day | 46 | -1.44 | (-2.48, -0.40), 0.007 | 46.9 | <0.001 | |  |
| > 10 gr/day | 46 | -1.31 | (-2.55, -0.08), 0.036 | 49.2 | <0.001 | |  |
| Gender |  |  |  |  |  | | **0.088** |
| Male | 8 | 0.17 | (-2.75, 3.11), 0.905 | 12.6 | 0.332 | |  |
| Female | 6 | -4.82 | (-10.29, 0.64), 0.084 | 69.1 | 0.006 | |  |
| Both | 77 | -1.23 | (-2.06, -0.39), 0.004 | 43.2 | <0.001 | |  |
| Baseline BMI |  |  |  |  |  | | **<0.001** |
| <30 | 56 | -1.94 | (-2.78, -1.67), <0.001 | 50.2 | 0.002 | |  |
| ≥30 | 27 | -1.26 | (-2.82, 0.29), 0.113 | 30.0 | 0.020 | |  |
| Age |  |  |  |  |  | | **<0.001** |
| < 50 y | 41 | -0.61 | (-1.86, 0.63), 0.336 | 54.1 | <0.001 | |  |
| ≥ 50 y | 49 | -2.16 | (-3.06, -1.27), <0.001 | 26.5 | 0.049 | |  |
| Baseline SBP | | | | | | | **<0.001** |
| Normal | 49 | -0.47 | (-1.33, 0.39), 0.285 | 21.9 | 0.091 | |  |
| Hypertention (≥130) | 45 | -2.53 | (-3.78, -1.29), 0.001 | 52.4 | <0.001 | |  |
| Fiber type |  |  |  |  |  | | **0.029** |
| Fermented | 78 | -1.38 | (-2.16, -0.59), 0.001 | 36.0 | 0.001 | |  |
| Non-fermented | 14 | -0.76 | (-3.39, 1.87), <0.001 | 72.6 | <0.001 | |  |
| Mix |  |  |  |  |  | |  |
| Fiber type | | | | | | | **0.567** |
| Viscose | 78 | -1.17 | (-1.99, -0.35), 0.005 | 49.1 | <0.001 | |  |
| Non-viscose | 14 | -2.43 | (-4.96, 0.10), 0.060 | 41.6 | 0.051 | |  |
| Fiber type | | | | | | | **0.038** |
| Inulin | 12 | -2.98 | (-6.25, 0.27), 0.073 | 47.2 | 0.036 | |  |
| Beta-glucan | 31 | -1.21 | (-2.48, 0.05), 0.060 | 33.9 | 0.871 | |  |
| Resistant starch | 9 | -1.04 | (-1.64, -0.45), 0.001 | 0.0 | <0.001 | |  |
| Psyllium | 14 | -0.76 | (-3.39, 1.87), 0.571 | 72.6 | 0.134 | |  |
| Guar gum | 9 | -2.25 | (-4.14, -0.37), 0.019 | 35.5 | 0.057 | |  |
| Konjac Glucomannan | 9 | -2.86 | (-6.35, 0.62), 0.108 | 47.1 | 0.042 | |  |
| Other | 10 | -0.03 | (-2.74, 2.67), 0.980 | 48.4 | <0.001 | |  |
| Health status | | | | | | | **<0.001** |
| Healthy | 9 | -1.34 | (-3.66, 0.79), 0.257 | 0.0 | 0.637 | |  |
| Hypercholesterolemia | 23 | -0.85 | (-2.33, 0.62),0.257 | 19.9 | 0.194 | |  |
| Hyperlipidemia | 2 | -0.61 | (-4.34, 3.10), 0.805 | 0.0 | 0.805 | |  |
| Diabetes | 22 | -2.50 | (-4.84, -0.16), 0.036 | 66.2 | <0.001 | |  |
| Overweight-Obese | 20 | 0.01 | (-1.89, 1.89), 0.999 | 30.7 | 0.095 | |  |
| Hypertension | 7 | -2.91 | (-4.08, -1.73), <0.001 | 18.4 | 0.289 | |  |
| Metabolic syndrome | 9 | -2.49 | (-4.29, -0.68), 0.007 | 42.8 | 0.082 | |  |
| Other diseases | 2 | -2.28 | (-13.73, 9.17), 0.037 | 77.0 | 0.037 | |  |
| **DBP (All trials)** | **93** | **-0.72** | **(-1.26, -0.18), 0.009** | **45.4** | **<0.001** |  | |
| Duration |  |  |  |  |  | | **0.037** |
| < 8 weeks | 39 | -0.46 | (-1.38, -0.46), 0.326 | 37.4 | 0.011 | |  |
| ≥ 8 weeks | 54 | -0.91 | (-1.57, -0.23), 0.008 | 48.8 | <0.001 | |  |
| Dose |  |  |  |  |  | | **0.003** |
| ≤ 10 gr/day | 45 | -0.54 | (-1.32, 0.23), 0.166 | 51.0 | <0.001 | |  |
| > 10 gr/day | 46 | -1.09 | (-1.85, -0.34), 0.004 | 32.2 | 0.021 | |  |
| Gender |  |  |  |  |  | | **0.018** |
| Male | 8 | 1.18 | (-1.05, 3.41), 0.299 | 25.2 | 0.228 | |  |
| Female | 6 | -2.01 | (-4.79, 0.77), 0.157 | 50.5 | 0.072 | |  |
| Both | 74 | -0.68 | (-1.33, -0.33), 0.039 | 46.1 | <0.001 | |  |
| Baseline BMI |  |  |  |  |  | | **0.684** |
| <30 | 54 | -0.76 | (-1.23, -0.25), 0.003 | 4.3 | 0.386 | |  |
| ≥30 | 28 | -1.62 | (-2.98, -0.27), 0.019 | 72.6 | <0.001 | |  |
| Age |  |  |  |  |  | | **0.080** |
| < 50 y | 41 | -0.52 | (-1.46, 0.41), 0.269 | 59.3 | <0.001 | |  |
| ≥ 50 y | 48 | -0.99 | (-1.61, -0.37), 0.002 | 21.2 | 0.102 | |  |
| Baseline DBP | | | | | | | **<0.001** |
| Normal | 46 | 0.28 | (-0.51, 1.08), 0.484 | 40.7 | 0.003 | |  |
| Hypertention (≥80 mmHg) | 47 | -1.56 | (-2.22, -0.91), <0.001 | 31.3 | 0.023 | |  |
| Fiber type |  |  |  |  |  | | **0.194** |
| Fermented | 77 | -0.81 | (-1.41, -0.21), 0.008 | 45.9 | <0.001 | |  |
| Non-fermented | 14 | 0.13 | (-1.29, 1.55), 0.861 | 47.3 | 0.026 | |  |
| Fiber type |  |  |  |  |  | | **<0.001** |
| Viscose | 76 | -0.34 | (-0.88, 0.20), 0.220 | 40.4 | <0.001 | |  |
| Non-viscose | 15 | -2.89 | (-4.62, -1.15), 0.001 | 42.1 | 0.043 | |  |
| Fiber type | | | | | | | **0.002** |
| Inulin | 13 | -3.20 | (-5.19, -1.22), 0.002 | 43.5 | 0.047 | |  |
| Beta-glucan | 29 | -0.49 | (-1.51, 0.53), 0.349 | 52.4 | 0.001 | |  |
| Resistant starch | 9 | -0.35 | (-0.86, 0.15), 0.173 | 0.0 | 0.441 | |  |
| Psyllium | 14 | 0.12 | (-1.29, 1.55), 0.861 | 47.3 | 0.026 | |  |
| Guar gum | 9 | -1.36 | (-2.70, 0.02), 0.054 | 30.4 | 0.175 | |  |
| Konjac Glucomannan | 8 | 0.10 | (-2.37, 2.58), 0.932 | 44.6 | 0.081 | |  |
| Other | 11 | -0.52 | (-1.75, 0.70), 0.401 | 2.2 | 0.421 | |  |
| Health status |  |  |  |  |  | | **<0.001** |
| Healthy | 9 | 0.22 | (-1.23, 1.69), 0.760 | 0.0 | 0.853 | |  |
| Hypercholesterolemia | 21 | -0.24 | (-1.06, 0.58), 0.562 | 0.0 | 0.641 | |  |
| Hyperlipidemia | 2 | 0.41 | (-3.18, 4.01), 0.822 | 0.0 | 0.405 | |  |
| Diabetes | 24 | -1.16 | (-2.34, 0.01), 0.051 | 41.2 | 0.019 | |  |
| Overweight-Obese | 18 | 0.11 | (-1.71, 1.95), 0.900 | 60.7 | <0.001 | |  |
| Hypertension | 7 | -2.10 | (-3.16, -1.03), <0.001 | 26.7 | 0.225 | |  |
| Metabolic syndrome | 9 | -1.12 | (-2.67, 0.42), 0.154 | 50.1 | 0.042 | |  |
| Other diseases | 3 | -2.94 | (-9.67, 3.78), 0.391 | 82.1 | 0.004 | |  |

^1^Calculated by Random-effects model.

**Abbreviations:** CI; Confidence Interval, SBP; , DBP;

| **Table S6**. Risk of bias assessment in randomised controlled trials | | | | | | | | |
| --- | --- | --- | --- | --- | --- | --- | --- | --- |
|  | **Random Sequence generation** | **Allocation**  **concealment** | **Blinding of**  **participants and**  **personnel** | **Blinding of outcome**  **assessment** | **Incomplete**  **outcome**  **data** | **selective outcome**  **reporting** | **Other**  **sources of**  **bias** | **Overall**  **Quality** |
| Momenizadeh (2014) (1) | L | U | U | U | L | L | U | Good |
| Jarrar (2021) (2) | L | U | H | U | H | L | U | Fair |
| Reimer (2020) (3) | L | U | L | U | L | L | U | Good |
| Zhang (2020) (4) | L | L | L | L | H | L | U | Good |
| Hiel (2020) (5) | L | L | H | U | L | L | U | Good |
| Cicero (2020)(6) | L | U | L | U | L | L | U | Good |
| Kitagawa (2019) (7) | L | L | L | U | L | L | U | Good |
| Peterson (2018) (8) | L | U | U | U | H | L | U | Fair |
| Vaghef-Mehrabany (2019) (9) | L | L | L | U | H | L | U | Good |
| Abbasalizad Farhangi (2019) (10) | L | L | U | U | H | L | U | Good |
| Lundby Hess (2019) (11) | L | L | L | U | H | L | U | Good |
| Abbasalizad Farhangi (2016) (12) | L | L | U | U | H | L | U | Good |
| Babiker (2018) (13) | L | U | L | U | L | L | U | Good |
| Gulati (2017) (14) | L | L | L | U | L | L | U | Good |
| Machado (2018) (15) | L | L | L | U | U | L | U | Good |
| Tabesh (2014 )(16) | L | L | L | L | U | L | U | Good |
| Sartore (2009) (17) | L | L | L | U | H | L | U | Good |
| Pal (2011) (18) | L | L | L | U | L | L | U | Good |
| de Souza (2019) (19) | L | U | H | U | L | L | U | Good |
| Abutair (2018) (20) | L | L | U | L | L | L | U | Good |
| Solà (2010) (21) | L | L | U | U | H | L | U | Good |
| Mosikanon (2016) (22) | L | L | U | U | L | L | U | Good |
| Dehghan (2016) (23) | U | L | U | U | L | L | U | Good |
| Chang (2013) (24) | L | L | U | U | U | L | U | Good |
| Lee (2017) (25) | L | U | L | U | L | L | U | Good |
| Jensen (2012) (26) | L | U | U | U | H | L | U | Fair |
| Davy (2002) (27) | L | U | U | L | L | L | U | Good |
| Wood (2007) (28) | U | U | L | U | L | L | U | Good |
| Tessari (2017) (29) | L | U | L | L | U | L | U | Good |
| Saltzman (2001) (30) | L | U | U | U | U | L | U | Fair |
| Dall’Alba (2013) (31) | L | U | H | H | L | L | U | Good |
| Anderson (1988) (32) | U | L | U | U | L | L | U | Good |
| Bell (1989) (33) | L | U | U | U | L | L | U | Good |
| Levin (1990) (34) | L | L | U | U | U | L | U | Good |
| Liatis (2009) (35) | L | L | U | U | L | L | U | Good |
| Makkonen (1993) (36) | L | U | U | U | L | L | U | Good |
| Maeda (2005) (37) | L | U | U | U | L | L | U | Good |
| J. Pins (2002) (38) | L | U | U | U | L | L | U | Good |
| Park (2004) (39) | L | U | U | U | L | L | U | Good |
| Azezli (2007) (40) | L | U | H | U | L | L | U | Good |
| Gómez-Reyes (2010) (41) | L | L | L | U | L | L | U | Good |
| Neyrinck (2021) (42) | L | U | H | U | U | L | U | Fair |
| Aoe (2017) (43) | L | U | L | U | L | L | U | Good |
| Liao (2018) (44) | L | L | U | U | H | L | U | Good |
| Reimer (2013) (45) | U | U | L | U | L | L | U | Good |
| Benítez-Páez (2021) (46) | L | L | L | U | U | L | U | Good |
| Vuksan (2000) (47) | L | U | U | U | L | L | U | Good |
| Zhang (2012) (48) | U | U | H | L | L | L | U | Good |
| Hokazono (2010) (49) | L | U | U | U | L | L | U | Good |
| Nishimura (2015) (50) | L | U | U | U | L | L | U | Good |
| Cai (2018) (51) | U | L | U | U | L | L | U | Good |
| Tuomilehto (1988) (52) | L | L | U | U | U | L | U | Good |
| Raimondi de Souza (2016) (53) | L | U | H | U | L | L | U | Good |
| Neal (1990) (54) | L | U | U | U | L | L | U | Good |
| Cheang (2017) (55) | L | U | U | U | H | L | U | Fair |
| Eshghi (2019) (56) | L | L | L | L | H | L | U | Good |
| Önning (1999) (57) | L | U | U | U | H | L | U | Fair |
| Arvill (1995) (58) | U | L | U | U | L | L | U | Good |
| Sakai (2019) (59) | U | L | U | U | L | L | U | Good |
| Uusitupa (1989) (60) | L | U | H | U | L | L | U | Good |
| Upadhyaya (2016) (61) | L | U | U | U | H | L | U | Fair |
| Jenkins (2002) (62) | L | U | U | U | H | L | U | Fair |
| Vuksan (1999) (63) | L | U | U | U | L | L | U | Good |
| Penn-Marshall (2010) (64) | L | U | U | U | L | L | U | Good |
| Nichenametla (2014) (65) | U | U | L | U | L | L | U | Good |
| Schwab (2006) (66) | L | U | U | U | L | L | U | Good |
| Lupton (1994) (67) | L | U | U | U | L | L | U | Good |
| Geliebter (2014) (68) | L | U | U | U | L | L | U | Good |
| Cicero (2007) (69) | L | L | L | U | U | L | U | Good |
| Bell (1990) (70) | U | U | L | U | L | L | U | Good |
| Chen (2016) (71) | L | U | L | U | L | L | U | Good |
| Charlton (2012) (72) | L | U | H | U | L | L | U | Good |
| Roshanravan (2017) (73) | L | U | U | U | L | L | U | Good |
| Cicero (2009) (74) | L | U | L | U | L | L | U | Good |
| Ghalandari (2018) (75) | L | U | L | U | L | L | U | Good |
| He (2004) (76) | L | L | L | U | H | L | U | Good |
| Ibrugger (2013) (77) | L | U | H | U | H | L | U | Fair |
| Tripkovic (2014) (78) | L | U | U | U | L | L | U | Good |
| Burke (2001) (79) | L | L | U | L | L | L | U | Good |
| Johnstone (2020) (80) | L | L | U | U | L | U | U | Good |
| Vuksan (2020) (81) | L | U | U | U | L | L | U | Good |
| Jenkins (1997) (82) | L | U | U | U | L | L | U | Good |
| Wong (2013) (83) | L | U | L | L | U | L | U | Good |

| **Table S7.** The GRADE evidence quality for each outcome | | | | | | | | | | | |
| --- | --- | --- | --- | --- | --- | --- | --- | --- | --- | --- | --- |
| Certainty assessment | | | | | | | No of patients | | Effect | Certainty | Importance |
| No of studies | Design | Risk of bias | Inconsistency | Indirectness | Imprecision | Other  considerations | Treatment group | Control group | MD (95%CI) |  |  |
| **SBP** | | | | | | | | | | | |
| 94 | randomised trials | not serious | not serious | serious^a^ | not serious | dose response gradient | 3006 | 2939 | MD -**1.36 mmHg lower** (2.13 lower to 0.60 higher) | ⨁⨁⨁◯ Moderate | IMPORTANT |
| **DBP** | | | | | | | | | | | |
| 93 | randomised trials | not serious | not serious | serious^a^ | not serious | dose response gradient | 2970 | 2897 | MD -**0.72 mmHg lower** (1,26 lower to 0.18 higher) | ⨁⨁⨁◯ Moderate | IMPORTANT |

MD; Mean Difference, CI; Confidence Interval.

Explanations

a. Serious indirectness since most of the trials were conducted in mixed populations. Downgraded.

**Supplementary References**

1. Momenizadeh A, Heidari R, Sadeghi M, Tabesh F, Ekramzadeh M, Haghighatian Z, et al. Effects of oat and wheat bread consumption on lipid profile, blood sugar, and endothelial function in hypercholesterolemic patients: a randomized controlled clinical trial. ARYA atherosclerosis. 2014;10(5):259.

2. Jarrar AH, Stojanovska L, Apostolopoulos V, Feehan J, Bataineh MaF, Ismail LC, et al. The effect of gum arabic (Acacia senegal) on cardiovascular risk factors and gastrointestinal symptoms in adults at risk of metabolic syndrome: A randomized clinical trial. Nutrients. 2021;13(1):194.

3. Reimer RA, Wharton S, Green TJ, Manjoo P, Ramay HR, Lyon MR, et al. Effect of a functional fibre supplement on glycemic control when added to a year-long medically supervised weight management program in adults with type 2 diabetes. European Journal of Nutrition. 2021;60(3):1237-51.

4. Zhang L, Han Y, Zhao Z, Liu X, Xu Y, Cui G, et al. Beneficial effects of konjac powder on lipid profile in schizophrenia with dyslipidemia: A randomized controlled trial. Asia Pacific Journal of Clinical Nutrition. 2020;29(3):505-12.

5. Hiel S, Gianfrancesco MA, Rodriguez J, Portheault D, Leyrolle Q, Bindels LB, et al. Link between gut microbiota and health outcomes in inulin-treated obese patients: Lessons from the Food4Gut multicenter randomized placebo-controlled trial. Clinical Nutrition. 2020;39(12):3618-28.

6. Cicero AF, Fogacci F, Veronesi M, Strocchi E, Grandi E, Rizzoli E, et al. A randomized placebo-controlled clinical trial to evaluate the medium-term effects of oat fibers on human health: the beta-glucan effects on lipid profile, glycemia and intestinal health (BELT) study. Nutrients. 2020;12(3):686.

7. Kitagawa M, Nakagawa S, Suzuki T, Kishimoto Y, Kanahori S, Hatakeyama Y, et al. Visceral fat-reducing effect and safety of continuous consumption of beverage containing resistant maltodextrin: A randomized, double-blind, placebo-controlled, parallel-group clinical trial. Journal of nutritional science and vitaminology. 2020;66(5):417-26.

8. Peterson CM, Beyl RA, Marlatt KL, Martin CK, Aryana KJ, Marco ML, et al. Effect of 12 wk of resistant starch supplementation on cardiometabolic risk factors in adults with prediabetes: a randomized controlled trial. The American journal of clinical nutrition. 2018;108(3):492-501.

9. Vaghef-Mehrabany E, Ranjbar F, Asghari-Jafarabadi M, Hosseinpour-Arjmand S, Ebrahimi-Mameghani M. Calorie restriction in combination with prebiotic supplementation in obese women with depression: Effects on metabolic and clinical response. Nutritional neuroscience. 2021;24(5):339-53.

10. Farhangi MA, Dehghan P, Namazi N. Prebiotic supplementation modulates advanced glycation end-products (AGEs), soluble receptor for AGEs (sRAGE), and cardiometabolic risk factors through improving metabolic endotoxemia: a randomized-controlled clinical trial. European journal of nutrition. 2020;59(7):3009-21.

11. Hess AL, Benítez-Páez A, Blædel T, Larsen LH, Iglesias JR, Madera C, et al. The effect of inulin and resistant maltodextrin on weight loss during energy restriction: a randomised, placebo-controlled, double-blinded intervention. European journal of nutrition. 2020;59(6):2507-24.

12. Farhangi MA, Javid AZ, Dehghan P. The effect of enriched chicory inulin on liver enzymes, calcium homeostasis and hematological parameters in patients with type 2 diabetes mellitus: a randomized placebo-controlled trial. Primary care diabetes. 2016;10(4):265-71.

13. Babiker R, Elmusharaf K, Keogh MB, Saeed AM. Effect of Gum Arabic (Acacia Senegal) supplementation on visceral adiposity index (VAI) and blood pressure in patients with type 2 diabetes mellitus as indicators of cardiovascular disease (CVD): a randomized and placebo-controlled clinical trial. Lipids in health and disease. 2018;17(1):1-8.

14. Gulati S, Misra A, Pandey RM. Effects of 3 g of soluble fiber from oats on lipid levels of Asian Indians-a randomized controlled, parallel arm study. Lipids in health and disease. 2017;16(1):1-8.

15. Machado AM, da Silva NB, Chaves JBP, Rita de Cássia GA. Consumption of yacon flour improves body composition and intestinal function in overweight adults: A randomized, double-blind, placebo-controlled clinical trial. Clinical nutrition ESPEN. 2019;29:22-9.

16. Tabesh F, Sanei H, Jahangiri M, Momenizadeh A, Tabesh E, Pourmohammadi K, et al. The effects of beta-glucan rich oat bread on serum nitric oxide and vascular endothelial function in patients with hypercholesterolemia. BioMed research international. 2014;2014.

17. Sartore G, Reitano R, Barison A, Magnanini P, Cosma C, Burlina S, et al. The effects of psyllium on lipoproteins in type II diabetic patients. European journal of clinical nutrition. 2009;63(10):1269-71.

18. Pal S, Khossousi A, Binns C, Dhaliwal S, Radavelli-Bagatini S. The effects of 12-week psyllium fibre supplementation or healthy diet on blood pressure and arterial stiffness in overweight and obese individuals. British journal of nutrition. 2012;107(5):725-34.

19. de Souza Leão LSC, de Aquino LA, Dias JF, Koifman RJ. Addition of oat bran reduces HDL-C and does not potentialize effect of a low-calorie diet on remission of metabolic syndrome: a pragmatic, randomized, controlled, open-label nutritional trial. Nutrition. 2019;65:126-30.

20. Abutair AS, Naser IA, Hamed AT. The effect of soluble fiber supplementation on metabolic syndrome profile among newly diagnosed type 2 diabetes patients. Clinical Nutrition Research. 2018;7(1):31-9.

21. Solà R, Bruckert E, Valls R-M, Narejos S, Luque X, Castro-Cabezas M, et al. Soluble fibre (Plantago ovata husk) reduces plasma low-density lipoprotein (LDL) cholesterol, triglycerides, insulin, oxidised LDL and systolic blood pressure in hypercholesterolaemic patients: a randomised trial. Atherosclerosis. 2010;211(2):630-7.

22. Mosikanon K, Arthan D, Kettawan A, Tungtrongchitr R, Prangthip P. Yeast β–glucan modulates inflammation and waist circumference in overweight and obese subjects. Journal of Dietary Supplements. 2017;14(2):173-85.

23. Dehghan P, Farhangi MA, Tavakoli F, Aliasgarzadeh A, Akbari AM. Impact of prebiotic supplementation on T-cell subsets and their related cytokines, anthropometric features and blood pressure in patients with type 2 diabetes mellitus: a randomized placebo-controlled trial. Complementary therapies in medicine. 2016;24:96-102.

24. Chang H-C, Huang C-N, Yeh D-M, Wang S-J, Peng C-H, Wang C-J. Oat prevents obesity and abdominal fat distribution, and improves liver function in humans. Plant foods for human nutrition. 2013;68(1):18-23.

25. Lee YJ, Paik D-J, Kwon DY, Yang HJ, Park Y. Agrobacterium sp.-derived β-1, 3-glucan enhances natural killer cell activity in healthy adults: a randomized, double-blind, placebo-controlled, parallel-group study. Nutrition research and practice. 2017;11(1):43-50.

26. Georg Jensen M, Kristensen M, Astrup A. Effect of alginate supplementation on weight loss in obese subjects completing a 12-wk energy-restricted diet: a randomized controlled trial. The American journal of clinical nutrition. 2012;96(1):5-13.

27. Davy BM, Melby CL, Beske SD, Ho RC, Davrath LR, Davy KP. Oat consumption does not affect resting casual and ambulatory 24-h arterial blood pressure in men with high-normal blood pressure to stage I hypertension. The Journal of nutrition. 2002;132(3):394-8.

28. Wood RJ, Fernandez ML, Sharman MJ, Silvestre R, Greene CM, Zern TL, et al. Effects of a carbohydrate-restricted diet with and without supplemental soluble fiber on plasma low-density lipoprotein cholesterol and other clinical markers of cardiovascular risk. Metabolism. 2007;56(1):58-67.

29. Tessari P, Lante A. A multifunctional bread rich in beta glucans and low in starch improves metabolic control in type 2 diabetes: a controlled trial. Nutrients. 2017;9(3):297.

30. Saltzman E, Das SK, Lichtenstein AH, Dallal GE, Corrales A, Schaefer EJ, et al. An oat-containing hypocaloric diet reduces systolic blood pressure and improves lipid profile beyond effects of weight loss in men and women. The Journal of nutrition. 2001;131(5):1465-70.

31. Dall'Alba V, Silva FM, Antonio JP, Steemburgo T, Royer CP, Almeida JC, et al. Improvement of the metabolic syndrome profile by soluble fibre–guar gum–in patients with type 2 diabetes: a randomised clinical trial. British Journal of Nutrition. 2013;110(9):1601-10.

32. Anderson JW, Zettwoch N, Feldman T, Tietyen-Clark J, Oeltgen P, Bishop CW. Cholesterol-lowering effects of psyllium hydrophilic mucilloid for hypercholesterolemic men. Archives of internal medicine. 1988;148(2):292-6.

33. Bell LP, Hectorne K, Reynolds H, Balm TK, Hunninghake DB. Cholesterol-lowering effects of psyllium hydrophilic mucilloid: adjunct therapy to a prudent diet for patients with mild to moderate hypercholesterolemia. Jama. 1989;261(23):3419-23.

34. Levin EG, Miller VT, Muesing RA, Stoy DB, Balm TK, LaRosa JC. Comparison of psyllium hydrophilic mucilloid and cellulose as adjuncts to a prudent diet in the treatment of mild to moderate hypercholesterolemia. Archives of internal medicine. 1990;150(9):1822-7.

35. Liatis S, Tsapogas P, Chala E, Dimosthenopoulos C, Kyriakopoulos K, Kapantais E, et al. The consumption of bread enriched with betaglucan reduces LDL-cholesterol and improves insulin resistance in patients with type 2 diabetes. Diabetes & metabolism. 2009;35(2):115-20.

36. Makkonen M, Simpanen A, Saarikoski S, Uusitupa M, Penttilä I, Silvasti M, et al. Endocrine and metabolic effects of guar gum in menopausal women. Gynecological endocrinology. 1993;7(2):135-41.

37. Maeda H, Yamamoto R, Hirao K, Tochikubo O. Effects of agar (kanten) diet on obese patients with impaired glucose tolerance and type 2 diabetes. Diabetes, obesity and metabolism. 2005;7(1):40-6.

38. O’Connor PJ, Cherney LM. Do whole-grain oat cereals reduce the need for antihypertensive medications and improve blood pressure control. J Fam Pract. 2002;51(4):353.

39. Park OJ, EKANG N, Chang MJ, Kim WK. Resistant starch supplementation influences blood lipid concentrations and glucose control in overweight subjects. Journal of nutritional science and vitaminology. 2004;50(2):93-9.

40. Azezli AD, Bayraktaroglu T, Orhan Y. The use of konjac glucomannan to lower serum thyroid hormones in hyperthyroidism. Journal of the American College of Nutrition. 2007;26(6):663-8.

41. Gómez-Reyes E, Orea-Tejeda A, Castillo-Martínez L, Cassis-Nosthas L, Vargas-Vorácková F. Prebiotics consumption modifies diastolic blood pressure, but does not affect serum lipids concentration in volunteers with ischemic heart disease. Energy (kcal). 2010;128:139.

42. Neyrinck AM, Rodriguez J, Zhang Z, Seethaler B, Sánchez CR, Roumain M, et al. Prebiotic dietary fibre intervention improves fecal markers related to inflammation in obese patients: Results from the Food4Gut randomized placebo-controlled trial. European journal of nutrition. 2021;60(6):3159-70.

43. Aoe S, Ichinose Y, Kohyama N, Komae K, Takahashi A, Abe D, et al. Effects of high β-glucan barley on visceral fat obesity in Japanese individuals: A randomized, double-blind study. Nutrition. 2017;42:1-6.

44. Liao M-Y, Shen Y-C, Chiu H-F, Ten S-M, Lu Y-Y, Han Y-C, et al. Down-regulation of partial substitution for staple food by oat noodles on blood lipid levels: A randomized, double-blind, clinical trial. journal of food and drug analysis. 2019;27(1):93-100.

45. Reimer RA, Yamaguchi H, Eller LK, Lyon MR, Gahler RJ, Kacinik V, et al. Changes in visceral adiposity and serum cholesterol with a novel viscous polysaccharide in Japanese adults with abdominal obesity. Obesity. 2013;21(9):E379-E87.

46. Benítez‐Páez A, Hess AL, Krautbauer S, Liebisch G, Christensen L, Hjorth MF, et al. Sex, food, and the gut microbiota: disparate response to caloric restriction diet with fiber supplementation in women and men. Molecular Nutrition & Food Research. 2021;65(8):2000996.

47. Vuksan V, Sievenpiper JL, Owen R, Swilley JA, Spadafora P, Jenkins D, et al. Beneficial effects of viscous dietary fiber from Konjac-mannan in subjects with the insulin resistance syndrome: results of a controlled metabolic trial. Diabetes care. 2000;23(1):9-14.

48. Zhang J, Li L, Song P, Wang C, Man Q, Meng L, et al. Randomized controlled trial of oatmeal consumption versus noodle consumption on blood lipids of urban Chinese adults with hypercholesterolemia. Nutrition Journal. 2012;11(1):1-8.

49. Hokazono H, Omori T, Yamamoto T, Akaoka I, Ono K. Effects of a fermented barley extract on subjects with slightly high serum uric acid or mild hyperuricemia. Bioscience, biotechnology, and biochemistry. 2010:1003051893-.

50. Nishimura M, Ohkawara T, Kanayama T, Kitagawa K, Nishimura H, Nishihira J. Effects of the extract from roasted chicory (Cichorium intybus L.) root containing inulin-type fructans on blood glucose, lipid metabolism, and fecal properties. Journal of Traditional and Complementary Medicine. 2015;5(3):161-7.

51. Cai X, Yu H, Liu L, Lu T, Li J, Ji Y, et al. Milk Powder Co‐Supplemented with Inulin and Resistant Dextrin Improves Glycemic Control and Insulin Resistance in Elderly Type 2 Diabetes Mellitus: A 12‐Week Randomized, Double‐Blind, Placebo‐Controlled Trial. Molecular nutrition & food research. 2018;62(24):1800865.

52. Tuomilehto J, Silvasti M, Aro A, Koistinen A, Karttunen P, Gref C-G, et al. Long term treatment of severe hypercholesterolaemia with guar gum. Atherosclerosis. 1988;72(2-3):157-62.

53. de Souza SR, de Oliveira GMM, Luiz RR, Rosa G. Effects of oat bran and nutrition counseling on the lipid and glucose profile and anthropometric parameters of hypercholesterolemia patients. Nutrición Hospitalaria. 2016;33(1):123-30.

54. Neal G, Balm T. Synergistic effects of psyllium in the dietary treatment of hypercholesterolemia. Southern medical journal. 1990;83(10):1131-7.

55. Cheang K-U, Chen C-M, Chen CO, Liang F-Y, Shih C-K, Li S-C. Effects of glucomannan noodle on diabetes risk factors in patients with metabolic syndrome: A double-blinded, randomized crossover controlled trial. J Food Nutr Res. 2017;5(8):622-8.

56. Eshghi F, Bakhshimoghaddam F, Rasmi Y, Alizadeh M. Effects of resistant starch supplementation on glucose metabolism, lipid profile, lipid peroxidation marker, and oxidative stress in overweight and obese adults: randomized, double-blind, crossover trial. Clinical Nutrition Research. 2019;8(4):318-28.

57. Önning G, Wallmark A, Persson M, Åkesson B, Elmståhl S, Öste R. Consumption of oat milk for 5 weeks lowers serum cholesterol and LDL cholesterol in free-living men with moderate hypercholesterolemia. Annals of Nutrition and Metabolism. 1999;43(5):301-9.

58. Arvill A, Bodin L. Effect of short-term ingestion of konjac glucomannan on serum cholesterol in healthy men. The American journal of clinical nutrition. 1995;61(3):585-9.

59. Sakai C, Abe S, Kouzuki M, Shimohiro H, Ota Y, Sakinada H, et al. A randomized placebo-controlled trial of an oral preparation of high molecular weight fucoidan in patients with type 2 diabetes with evaluation of taste sensitivity. Yonago acta medica. 2019;62(1):014-23.

60. Uusitupa M, Siitonen O, Savolainen K, Silvasti M, Penttilä I, Parviainen M. Metabolic and nutritional effects of long-term use of guar gum in the treatment of noninsulin-dependent diabetes of poor metabolic control. The American journal of clinical nutrition. 1989;49(2):345-51.

61. Upadhyaya B, McCormack L, Fardin-Kia AR, Juenemann R, Nichenametla S, Clapper J, et al. Impact of dietary resistant starch type 4 on human gut microbiota and immunometabolic functions. Scientific reports. 2016;6(1):1-12.

62. Jenkins DJ, Kendall CW, Vuksan V, Vidgen E, Parker T, Faulkner D, et al. Soluble fiber intake at a dose approved by the US Food and Drug Administration for a claim of health benefits: serum lipid risk factors for cardiovascular disease assessed in a randomized controlled crossover trial. The American journal of clinical nutrition. 2002;75(5):834-9.

63. Vuksan V, Jenkins D, Spadafora P, Sievenpiper JL, Owen R, Vidgen E, et al. Konjac-mannan (glucomannan) improves glycemia and other associated risk factors for coronary heart disease in type 2 diabetes. A randomized controlled metabolic trial. Diabetes care. 1999;22(6):913-9.

64. Penn-Marshall M, Holtzman GI, Barbeau WE. African Americans may have to consume more than 12 grams a day of resistant starch to lower their risk for type 2 diabetes. Journal of medicinal food. 2010;13(4):999-1004.

65. Nichenametla SN, Weidauer LA, Wey HE, Beare TM, Specker BL, Dey M. Resistant starch type 4‐enriched diet lowered blood cholesterols and improved body composition in a double blind controlled cross‐over intervention. Molecular nutrition & food research. 2014;58(6):1365-9.

66. Schwab U, Louheranta A, Törrönen A, Uusitupa M. Impact of sugar beet pectin and polydextrose on fasting and postprandial glycemia and fasting concentrations of serum total and lipoprotein lipids in middle-aged subjects with abnormal glucose metabolism. European journal of clinical nutrition. 2006;60(9):1073-80.

67. Lupton JR, Robinson MC, Morin JL. Cholesterol-lowering effect of barley bran flour and oil. Journal of the American Dietetic Association. 1994;94(1):65-70.

68. Geliebter A, Astbury NM, Aviram-Friedman R, Yahav E, Hashim S. Skipping breakfast leads to weight loss but also elevated cholesterol compared with consuming daily breakfasts of oat porridge or frosted cornflakes in overweight individuals: a randomised controlled trial. Journal of Nutritional Science. 2014;3.

69. Cicero AF, Derosa G, Manca M, Bove M, Borghi C, Gaddi AV. Different effect of psyllium and guar dietary supplementation on blood pressure control in hypertensive overweight patients: a six-month, randomized clinical trial. Clinical and experimental hypertension. 2007;29(6):383-94.

70. Bell LP, Hectorn KJ, Reynolds H, Hunninghake DB. Cholesterol-lowering effects of soluble-fiber cereals as part of a prudent diet for patients with mild to moderate hypercholesterolemia. The American journal of clinical nutrition. 1990;52(6):1020-6.

71. Chen C, Zeng Y, Xu J, Zheng H, Liu J, Fan R, et al. Therapeutic effects of soluble dietary fiber consumption on type 2 diabetes mellitus. Experimental and therapeutic medicine. 2016;12(2):1232-42.

72. Charlton KE, Tapsell LC, Batterham MJ, O'Shea J, Thorne R, Beck E, et al. Effect of 6 weeks' consumption of β-glucan-rich oat products on cholesterol levels in mildly hypercholesterolaemic overweight adults. British Journal of Nutrition. 2012;107(7):1037-47.

73. Roshanravan N, Mahdavi R, Alizadeh E, Jafarabadi MA, Hedayati M, Ghavami A, et al. Effect of butyrate and inulin supplementation on glycemic status, lipid profile and glucagon-like peptide 1 level in patients with type 2 diabetes: a randomized double-blind, placebo-controlled trial. Hormone and metabolic research. 2017;49(11):886-91.

74. Cicero AF, Derosa G, Bove M, Imola F, Borghi C, Gaddi AV. Psyllium improves dyslipidaemia, hyperglycaemia and hypertension, while guar gum reduces body weight more rapidly in patients affected by metabolic syndrome following an AHA Step 2 diet. Mediterranean Journal of Nutrition and Metabolism. 2010;3(1):47-54.

75. Ghalandari H, Kamalpour M, Alimadadi A, Nasrollahzadeh J. Comparison of two calorie-reduced diets of different carbohydrate and fiber contents and a simple dietary advice aimed to modify carbohydrate intake on glycemic control and inflammatory markers in type 2 diabetes: a randomized trial. International journal of endocrinology and metabolism. 2018;16(1).

76. He J, Streiffer RH, Muntner P, Krousel-Wood MA, Whelton PK. Effect of dietary fiber intake on blood pressure: a randomized, double-blind, placebo-controlled trial. Journal of Hypertension. 2004;22(1):73-80.

77. Ibrügger S, Kristensen M, Poulsen MW, Mikkelsen MS, Ejsing J, Jespersen BM, et al. Extracted oat and barley β-glucans do not affect cholesterol metabolism in young healthy adults. The Journal of nutrition. 2013;143(10):1579-85.

78. Tripkovic L, Muirhead N, Hart K, Frost G, Lodge J. The effects of a diet rich in inulin or wheat fibre on markers of cardiovascular disease in overweight male subjects. Journal of Human Nutrition and Dietetics. 2015;28(5):476-85.

79. Burke V, Hodgson JM, Beilin LJ, Giangiulioi N, Rogers P, Puddey IB. Dietary protein and soluble fiber reduce ambulatory blood pressure in treated hypertensives. Hypertension. 2001;38(4):821-6.

80. Johnstone AM, Kelly J, Ryan S, Romero-Gonzalez R, McKinnon H, Fyfe C, et al. Nondigestible carbohydrates affect metabolic health and gut microbiota in overweight adults after weight loss. The Journal of Nutrition. 2020;150(7):1859-70.

81. Vuksan V, Sievenpiper JL, Jovanovski E, Jenkins AL, Komishon A, Au-Yeung F, et al. Effect of soluble-viscous dietary fibre on coronary heart disease risk score across 3 population health categories: data from randomized, double-blind, placebo-controlled trials. Applied Physiology, Nutrition, and Metabolism. 2020;45(7):801-4.

82. Jenkins D, Wolever T, Vidgen E, Kendall C, Ransom T, Mehling C, et al. Effect of psyllium in hypercholesterolemia at two monounsaturated fatty acid intakes. The American journal of clinical nutrition. 1997;65(5):1524-33.

83. Wong RH, Howe PR, Coates AM, Buckley JD, Berry NM. Chronic consumption of a wild green oat extract (Neuravena) improves brachial flow-mediated dilatation and cerebrovascular responsiveness in older adults. Journal of Hypertension. 2013;31(1):192-200.
